# Effect of Fenofibrate on Progression of Diabetic Retinopathy

**DOI:** 10.1056/EVIDoa2400179

**Published:** 2024-06-21

**Authors:** David Preiss, Jennifer Logue, Emily Sammons, Mohammed Zayed, Jonathan Emberson, Rachel Wade, Karl Wallendszus, Will Stevens, Rosanna Cretney, Simon Harding, Graham Leese, Gemma Currie, Jane Armitage, for the LENS Collaborative Group

**Affiliations:** 1 Clinical Trial Service Unit and Epidemiological Studies Unit, Nuffield Department of Population Health, University of Oxford, Oxford; 2 Faculty of Health and Medicine, University of Lancaster, Lancaster; 3 Department of Eye and Vision Science, University of Liverpool and St. Paul’s Eye Unit, Liverpool University Hospitals NHS Foundation Trust, Liverpool; 4 Molecular and Clinical Medicine, University of Dundee, Dundee; 5 School of Cardiovascular & Metabolic Health, University of Glasgow, Glasgow

## Abstract

**Background:**

Findings from cardiovascular outcome trials suggest that fenofibrate therapy may reduce the progression of diabetic retinopathy.

**Methods:**

We recruited and followed adults with non-referable diabetic retinopathy or maculopathy using the national Diabetic Eye Screening (DES) Programme in Scotland. We randomized participants to receive fenofibrate 145mg tablets or placebo (taken daily or, in those with impaired renal function, on alternate days). The primary outcome was a composite of developing referable diabetic retinopathy or maculopathy (based on Scotland’s DES grading scheme), or treatment (intra-vitreal injection, retinal laser, vitrectomy) for retinopathy or maculopathy.

**Results:**

A total of 1151 participants underwent randomization. During a median of 4.0 years, progression to referable diabetic retinopathy or maculopathy, or treatment thereof, occurred in 131 (22.7%) of 576 participants in the fenofibrate group and 168 (29.2%) of 575 in the placebo group (hazard ratio [HR] 0.73; 95% confidence interval [CI] 0.58-0.91; P=0.006). In the fenofibrate group compared to the placebo group, the frequencies were lower for any progression of retinopathy or maculopathy (185 [32.1%] vs. 231 [40.2%]; HR 0.74; 95%CI 0.61-0.90) and for the development of macular edema (22 [3.8%] vs. 43 [7.5%]; HR 0.50; 95%CI 0.30-0.84). 17 (3.0%) participants assigned fenofibrate and 28 (4.9%) assigned placebo were given specific treatment for retinopathy (HR 0.58; 95%CI 0.31-1.06). There was no effect on visual function, quality of life or visual acuity. Trial-averaged estimated glomerular filtration rate was 7.9 (95%CI 6.8-9.1) mL/min/1.73m^2^ lower in participants in the fenofibrate group compared to the placebo group. Serious adverse event occurred in 208 (36.1%) participants allocated fenofibrate and 204 (35.5%) participants allocated placebo.

**Conclusions:**

Fenofibrate reduced progression of diabetic retinopathy compared to placebo among participants with early retinal changes.

**Trial registration:**

NCT03439345; ISRCTN15073006

Diabetic retinopathy is a common complication of diabetes mellitus and a leading cause of visual loss.^1^ Progression to proliferative retinopathy may cause vitreous hemorrhage, retinal detachment and neovascular glaucoma, resulting in visual impairment. Diabetic maculopathy is characterized by exudates, microaneurysms, and/or hemorrhages, which may progress to macular edema and also threaten vision. Treatments for advanced disease, like retinal laser and intra-vitreal injections, require expertise to deliver, are expensive, associated with iatrogenic risks, and sometimes ineffective.^2^ Cost-effective therapies that are efficacious earlier in the course of the disease are therefore needed. Population-based diabetic retinal screening is recommended to identify people at risk of vision-threatening disease^3^, and may also facilitate earlier intervention in those with non-vision-threatening disease.

Fenofibrate is an orally administered peroxisome proliferator-activated receptor alpha agonist that reduces circulating triglycerides and non-HDL cholesterol levels.^4^ In the Fenofibrate Intervention and Event Lowering in Diabetes (FIELD) trial, conducted in participants with type 2 diabetes, the tertiary outcome of retinal laser therapy for proliferative retinopathy or macular edema was reduced over five years.^5,6^ Retinal imaging sub-studies of FIELD and the Action to Control Cardiovascular Risk in Diabetes (ACCORD) Lipid trial have further supported the hypothesis that fenofibrate therapy may reduce diabetic retinopathy progression.^6–8^

Given that these results emerged from subsidiary outcomes in cardiovascular trials which did not show cardiovascular benefit, there is a need to conduct trials specifically designed to investigate the ocular effects of fenofibrate. The Lowering Events in Non-proliferative retinopathy in Scotland (LENS) trial – a pragmatic, national, randomized, parallel-group, double-masked, placebo-controlled, clinical trial of fenofibrate set within a national retinal screening programme – was designed to assess its effect on progression of retinopathy in people with early diabetic eye disease.^9^

## Methods

### Trial design and oversight

LENS was initiated by investigators at the Central Coordinating Office (CCO) at the Nuffield Department of Population Health (NDPH), University of Oxford (the trial sponsor). It was conducted at 16 sites in all 11 National Health Service (NHS) Health Boards in mainland Scotland and designed to be embedded within routine clinical care. Characteristics of the participants and trial methods have been reported previously.^9^ The protocol was approved by the West of Scotland Research Ethics Committee. The trial was primarily funded by the National Institute for Health and Care Research’s Health Technology Assessment Programme. Fenofibrate and matching placebo were provided by Mylan. The trial has oversight from a Steering Committee that includes patient representatives. An independent Data Monitoring Committee regularly reviewed unmasked data to ensure patient safety. All results presented herein derive from analyses conducted by statisticians at the CCO. D.P. wrote the first draft and all member of the Writing Committee contributed to revisions thereof. All authors vouch for the accuracy and completeness of the data and analyses, and for the fidelity of the trial to the protocol and the Data Analysis Plan (supplementary materials).

### Participants

Adults (aged ≥18 years) with diabetes mellitus and non-referable diabetic retinopathy or maculopathy were potentially eligible to join the trial. Non-referable disease was defined according to NHS Scotland’s Diabetic Eye Screening (DES) Programme grading scheme (Supplementary Appendix Table S1) as: (i) mild background retinopathy in both eyes or observable background retinopathy in one/both eyes at the most recent retinal screening assessment; or (ii) observable maculopathy in one/both eyes at a retinal screening assessment in the last three years (though participants were invited based on non-referable disease at their most recent retinal screening). Estimated glomerular function rate (eGFR) was required to be ≥40 mL/min/1.73m^2^ at the screening assessment. Full details regarding the eligibility criteria are provided in the protocol.^9^ All participants provided written informed consent.

### Trial procedures

Regular retinal screening is routinely offered to people with diabetes in Scotland by the national DES Programme.^10^ Single macula-centered 45-degree digital retinal color photographs of each eye showing the macula and optic disc are taken with non-mydriatic cameras using a staged imaging protocol.^11^ More details of the DES programme are provided in the Supplementary Appendix. Digital retinal images are graded according to the NHS Scotland DES Programme grading scheme (Supplementary Appendix Table S1) in ten grading centres. This approach has been validated for detection of referable disease requiring regular ophthalmologic review.^12,13^ Image graders participate in an annual quality assurance programme.^9^ Scottish Care Information – Diabetes (SCI Diabetes) is NHS Scotland’s national integrated electronic diabetes patient record, used in general practice and hospitals to support diabetes management, including the collection of biochemistry and retinal screening data.^14^ These and other national datasets supported streamlined recruitment and follow up of trial participants.

Adults across Scotland with non-referable diabetic retinopathy or maculopathy were invited to join the trial.^9^ Site staff performed pre-screening for interested respondents. Eligibility, including eGFR, was checked at an in-person trial screening visit following which participants entered an active pre-randomization run-in and were mailed a 10-week supply of open-label nanoparticle fenofibrate 145mg tablets. Those with screening eGFR ≥60 mL/min/1.73m^2^ commenced one tablet daily, and those with eGFR 40-59 mL/min/1.73m^2^ commenced one tablet on alternate days. Approximately eight weeks later, participants attended an in-person randomization assessment. At the randomization assessment the eGFR was required to be ≥30 mL/min/1.73m^2^ (lower than the threshold at the screening assessment) to allow for the anticipated increase in serum creatinine due to fenofibrate treatment during the run-in.^15,16^ During the COVID-19 pandemic, some randomization assessments were conducted by telephone and eGFR was checked as soon as possible thereafter to confirm eligibility. Participants were then randomized by a web-based algorithm to receive nanoparticle fenofibrate 145mg or matching placebo in a 1:1 ratio. Randomization was performed with the use of a minimization process (based on categories of sex, age, type of diabetes, eGFR at randomization, HbA1c, use of statin, most recent maculopathy grade, and most recent retinopathy grade) including a 10% stochastic element. The dose frequency depended on renal function. Participants with a randomization assessment eGFR ≥60 mL/min/1.73m^2^ commenced one tablet daily and those with eGFR 30-59 mL/min/1.73m^2^ commenced one tablet on alternate days. During follow up, participants continued DES retinal screening and were contacted by telephone every six months (with an option for follow up via medical record review or contact with their usual doctor if they were not contactable or declined further contact). Participants were regularly mailed supplies of randomized trial treatment. eGFR results from routine care were monitored centrally via SCI Diabetes. If post-randomization eGFR fell to 30-59 mL/min/1.73m^2^, trial treatment was reduced to one tablet on alternate days and, if below 30 mL/min/1.73m^2^, trial treatment was discontinued but could be recommenced if renal function subsequently improved. Linkages to DES and other national healthcare datasets were performed to identify certain pre-specified outcomes. The protocol required reporting of adverse events representing clinical trial outcomes, all serious adverse events and those adverse events (serious and non-serious) that led to stopping trial treatment.

A trial treatment mailing error, temporarily affecting 28 participants, occurred in late 2022 (details are provided in the Supplementary Appendix).

### Outcomes

The primary outcome was time to the first occurrence of the composite of developing referable diabetic retinopathy or maculopathy, or treatment for diabetic retinopathy or maculopathy (including intra-vitreal injection of medication, retinal laser therapy or vitrectomy) in either eye. Referable diabetic retinopathy or maculopathy was defined according to NHS Scotland’s DES Programme grading scheme (Supplementary Appendix Table S1) as: referable background (i.e. moderately severe or severe non-proliferative) diabetic retinopathy, or proliferative diabetic retinopathy, or referable maculopathy (any blot haemorrhage or exudate within 1 disc diameter distance of the foveal centre). Secondary outcomes included time to any progression of diabetic retinopathy or maculopathy; the development of referable maculopathy alone; the development of macular edema (including centre-involving and non-centre-involving macular edema as identified during slit lamp examination or optical coherence tomography); change in visual function (based on Visual Function Questionnaire-25 data), quality of life (based on EQ-5D 5-dimension questionnaire data) and visual acuity; components of the primary outcome (namely development of referable diabetic retinopathy or maculopathy; and treatment thereof); and the primary outcome in six pre-specified subgroups, namely men vs. women, age <60 vs. ≥60 years, type 1 diabetes vs. type 2 diabetes and other types, randomisation eGFR <60 vs. ≥60 mL/min/1.73m^2^, HbA1c <70 mmol/mol vs. ≥70 mmol/mol, and the timing of the primary outcome (first year after randomization vs. later years). Adverse event reports of eye procedures, vitreous hemorrhages and macular edema were adjudicated by doctors at the CCO, masked to treatment allocation. Referable retinopathy or maculopathy was typically identified during DES retinal screening but could also be identified during adjudication of adverse events.

Details of tertiary and other outcomes are provided in the Data Analysis Plan. Health economic analyses, although gathered, are not reported herein.

### Statistical analysis

NHS Scotland DES Programme data suggested that progression from non-referable to referable eye disease in the target population would occur in approximately 29% of individuals over four years.^9^ We calculated that a sample size of 1060 participants would provide 85% power (at 2-sided alpha of 0.05) to detect a 33% proportional reduction in the primary outcome, based on 222 first events occurring over four years, allowing for 15% drop-out (e.g. no longer attending retinal screening). The trial was designed to continue until both: (i) at least 222 primary outcome events had occurred; and (ii) at least four years had elapsed after randomization of the median participant. The Data Analysis Plan was finalized by the Steering Committee while members remained unaware of the unmasked results.

For time-to-event analyses, Cox proportional hazards regression analyses, adjusted for baseline covariates used for minimized randomization, were used to estimate the hazard ratios [HR], 95% confidence intervals and the corresponding p-value for the primary outcome, comparing participants allocated fenofibrate with those allocated placebo. The proportional hazards assumption was assessed by testing for a non-zero slope of the Schoenfeld residuals as a function of follow-up time. The unit of analysis was the participant (i.e. data from either eye could contribute). For continuous measures with baseline and repeated follow-up values, linear mixed model repeated measures (MMRM) analyses were conducted to estimate the mean values by treatment allocation at each follow-up time point, as well as a trial-averaged difference in mean follow-up levels between the randomized treatment groups. The MMRM models adjusted for the baseline value of the relevant measure, as well as the baseline minimization criteria, and assumed an unstructured covariance matrix. Analyses compared outcomes from randomization to the end of the scheduled treatment period among all participants allocated at randomization to receive fenofibrate versus placebo (i.e. “intention-to-treat” analyses). We did not define a testing hierarchy for the secondary outcomes so a p-value (2-sided) is only reported for the primary outcome and, since confidence intervals have not been adjusted for multiplicity, these should not be used to infer clinical utility. NDPH at the University of Oxford holds the full database. NDPH staff performed all analyses using SAS software, version 9.4 (SAS Institute) and figures were constructed using R software, versions 4.3.2 and 4.3.3.

## Results

### Trial participants

Of 1633 participants screened in the trial, 1484 entered the pre-randomization run-in and commenced open-label fenofibrate (Supplementary Appendix Figure S1). Information regarding serious adverse events during the run-in is summarized in Table S2 (Supplementary Appendix). One participant on concomitant simvastatin therapy was hospitalized for rhabdomyolysis during the run-in and was not randomized. From September 2018 through July 2021, 1151 participants underwent randomization. Key prognostic characteristics were well balanced between the randomized groups (Table 1). The mean age of participants was 61 years, 73% were men, 26% had type 1 diabetes, mean duration of diabetes was 18 years and mean HbA1c was 66 mmol/mol (8.2%). The substantial majority (96%) had bilateral mild background retinopathy, and 10% had observable maculopathy in at least one eye. The proportion with eGFR <60 mL/min/1.73m^2^ increased from 9% at the screening assessment to 23% at the randomization assessment, which was attributed to the effect of fenofibrate. Information regarding representativeness of the LENS trial participants is provided in Table S3.

At the end of the scheduled treatment period, complete data were available for 1149 randomized participants (99.8%) (Supplementary Appendix Table S4). Median follow-up until the end of the scheduled treatment period was 4.0 (interquartile range 3.6 to 4.3) years. The frequency of DES retinal screening was similar in both groups after randomization (Supplementary Appendix Table S5). Further details about retinal screening during the trial, including image quality and the use of mydriasis, are provided in Supplementary Appendix Table S6.

### Adherence to trial treatment

Average self-reported adherence to the assigned trial treatment regimen was similar between the groups (88% for fenofibrate and 89% for placebo). More participants assigned daily treatment with fenofibrate transitioned to alternate day treatment than placebo due to the effect of fenofibrate on eGFR (Supplementary Appendix Table S7). There were no differences in the number of participants who discontinued fenofibrate compared with placebo overall or for any specific reason (Supplementary Appendix Table S8).

### Effects on the primary outcome and secondary outcomes

**Figure 1:**
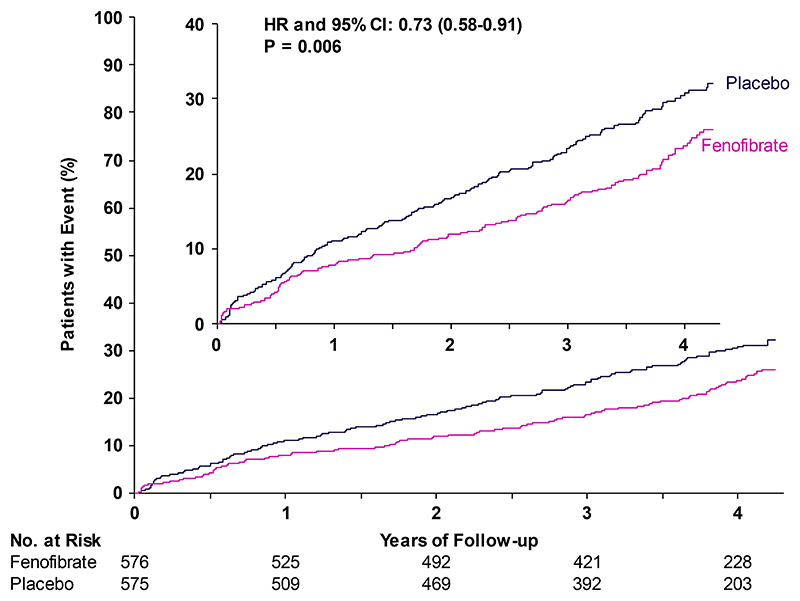
Referable diabetic retinopathy or maculopathy, or treatment for diabetic retinopathy or maculopathy Shown are the results of the primary composite outcome of referable diabetic retinopathy or maculopathy, or treatment thereof, with intra-vitreal injection of medication, retinal laser therapy or vitrectomy. The primary outcome occurred in 131 participants (22.7%) in the fenofibrate group and in 168 participants (29.2%) in the placebo group, representing 65 fewer primary outcome events per 1000 participants in the fenofibrate group than in the placebo group over a median of 4.0 years of follow-up.

During the scheduled treatment period, the primary outcome occurred in significantly fewer participants in the fenofibrate group than in the placebo group (131 [22.7%] vs. 168 [29.2%]; hazard ratio 0.73; 95% CI 0.58-0.91; P=0.006) (Figure 1), representing an absolute reduction of 6.5 percentage points (95% CI 1.4-11.5 percentage points) over a median of 4.0 years.

**Figure 2:**
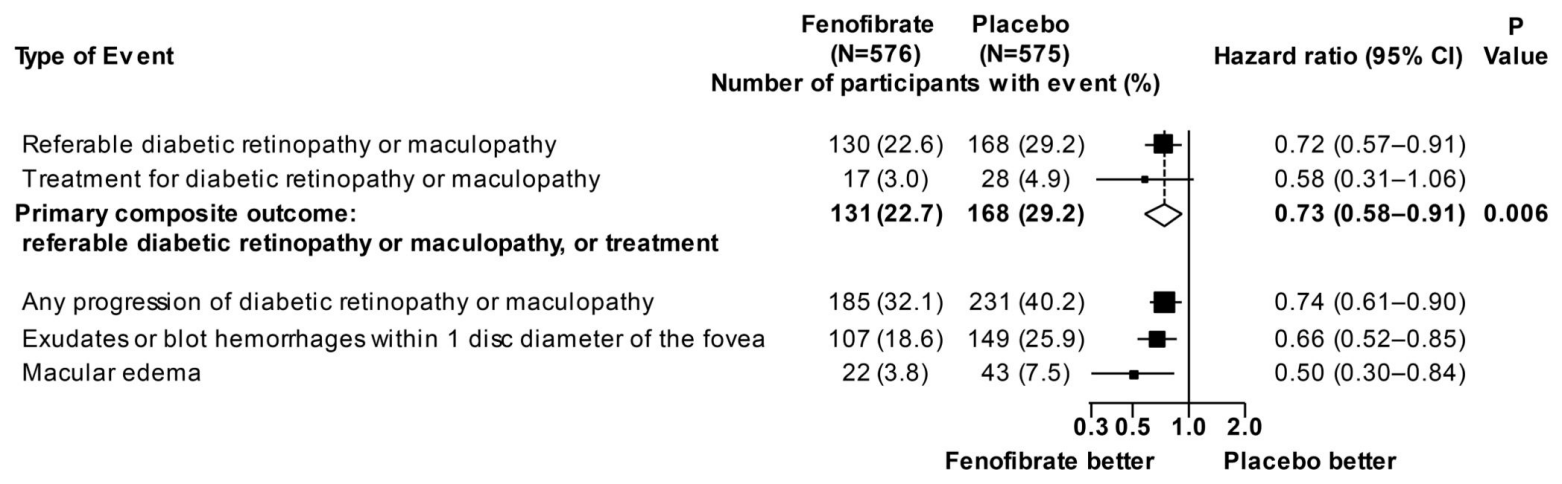
Primary and secondary eye outcomes The primary outcome was a composite of developing referable diabetic retinopathy or maculopathy, or treatment thereof, with intra-vitreal injection of medication, retinal laser therapy or vitrectomy. Secondary retinopathy outcomes were any progression of diabetic retinopathy or maculopathy; development of exudates or blot hemorrhages within one disc diameter of the macula; development of macular edema; and components of the primary outcome (namely referable diabetic retinopathy or maculopathy; and treatment for diabetic retinopathy or maculopathy). A single participant may have had multiple events and therefore may contribute information to more than one row. The area of each box is proportional to the inverse of the variance of the log hazard ratios. The diamond represents the result of the primary analysis, with the width of the diamond indicating the 95% confidence interval. The dashed vertical line indicates the hazard ratio for the primary outcome in the overall population. Confidence intervals for the secondary eye outcomes have not been adjusted for multiplicity, and should not be used to infer clinical utility. These results exclude adverse events not related to diabetic retinopathy or maculopathy, namely: macular edema (2 participants) and treatment with laser, intravitreal injection or vitrectomy (6 participants) in the fenofibrate arm, and macular edema (3 participants) and treatment (4 participants) in the placebo arm due to macular degeneration or retinal vein occlusion or a dropped lens.

Any retinopathy or maculopathy progression occurred in 185 participants in the fenofibrate group [32.1%] and 231 participants [40.2%] in the placebo group (hazard ratio 0.74; 95% CI 0.61-0.90). A similar proportional reduction was observed for referable maculopathy, affecting 107 (18.6%) participants allocated fenofibrate and 149 (25.9%) participants allocated placebo (hazard ratio 0.66; 95% CI 0.52-0.85) (Figure 2). Macular edema occurred in 22 [3.8%] participants in the fenofibrate group and 43 [7.5%] in the placebo group (hazard ratio 0.50; 95% CI 0.30-0.84) (Figure 2). Results for the components of the primary outcome were consistent with the overall result (Figure 2, Table S9). There was no effect of allocation to fenofibrate compared to placebo on visual function, quality of life or visual acuity (Supplementary Appendix Table S10).

**Figure 3:**
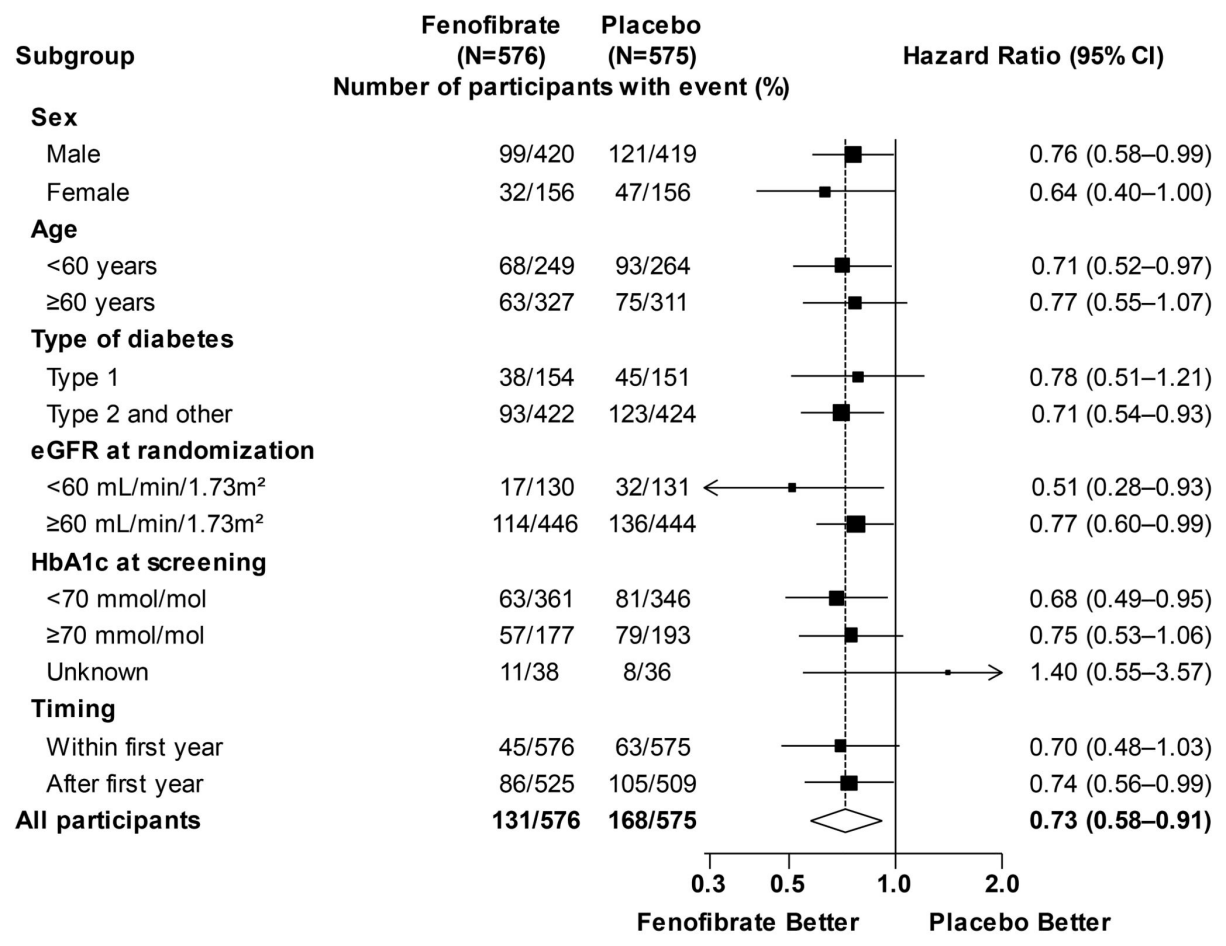
Primary outcome in pre-specified subgroups Shown are the hazard ratios for the primary outcome in prespecified subgroups defined according to baseline characteristics. The area of each box is proportional to the inverse of the variance of the log hazard ratios. The arrow indicates that the boundary of the 95% confidence interval is outside the graphed area. The diamond represents the result of the primary analysis, with the width of the diamond indicating the 95% confidence interval. The dashed line indicates the hazard ratio in the overall population.

There was no evidence of differential proportional effects within pre-specified subgroup categories, including type 1 vs. type 2 and other types of diabetes, and normal vs. impaired renal function (Figure 3).

### Effects on other efficacy, biochemical and safety outcomes

In the population of all participants allocated to receive trial treatment, there was no difference in the occurrence of major cardiovascular events or non-traumatic lower limb amputation in the fenofibrate group compared to the placebo group (Supplementary Appendix Table S11), and no effect on urine albumin creatinine ratio (Table 2, Supplementary Appendix Table S12).

The eGFR was 7.9 mL/min/1.73m^2^ lower in the fenofibrate group than the placebo group on average (Table 2), with similar results during each year (Figure S2, Supplementary Appendix Table S12). Total cholesterol, non-HDL cholesterol and triglycerides in participants allocated fenofibrate compared to placebo are given in Table 2 and Supplementary Appendix Table S12; absolute differences in these measures were small except for triglycerides for which the absolute mean reduction was 22.2 mg/dl in the fenofibrate group with no change in the control group, a 13.7% difference.

Overall, 35 (6.1%) participants allocated fenofibrate and 38 (6.6%) participants allocated placebo died. Rates of fatal and non-fatal serious adverse events, grouped according to Medical Dictionary for Regulatory Activities system organ class, were similar between treatment groups (Supplementary Appendix Table S13).

## Discussion

In this population of people with diabetes and early retinopathy, fenofibrate led to a 27% lower risk of progression of, or treatment for, diabetic retinopathy or maculopathy compared with placebo over 4 years. Treatment with fenofibrate appeared similarly effective in participants with type 1 and type 2 diabetes, and those with normal and or somewhat impaired kidney function.

The proportional effect of fenofibrate on the progression of retinopathy or maculopathy observed in LENS is quantitatively similar to that seen in subsidiary analyses from major cardiovascular trials. In the FIELD trial, the tertiary outcome of retinal laser therapy was proportionally reduced by 31% (164 [3.4%] events in 4895 participants allocated fenofibrate vs. 238 [4.9%] events in 4900 participants allocated placebo) over five years, and pooled data from three trials showed a 23% reduction in retinal laser compared to placebo.^5,6,8^ A FIELD imaging sub-study showed a 34% proportional reduction in a post-hoc composite outcome of two-step Early Treatment Diabetic Retinopathy Study (ETDRS) Severity Scale retinopathy progression, new macular edema or retinal laser (53 [11.1%] events in 512 participants vs. 75 [16.1%] events in 500 participants on placebo). In addition, the ACCORD Eye sub-study yielded a 40% proportional reduction in the composite outcome of three-step ETDRS retinopathy progression, retinal laser or vitrectomy (52 [6.5%] events in 806 participants vs. 80 [10.2%] events in 787 participants on placebo) over four years.^6,7^

Fenofibrate therapy led to a trial-averaged reduction in eGFR of 8 mL/min/1.73m^2^ compared to placebo. Fenofibrate is known to reversibly increase blood creatinine levels though there is also some evidence that it may offer renal protection.^15,16^ The fall in eGFR is clinically relevant given that the recommended dose of fenofibrate depends on eGFR and because it may complicate interpretation of eGFR results in people with diabetes, a group at elevated risk of developing chronic kidney disease. We observed no benefit on visual acuity, visual function or quality of life. This may reflect the generally mild retinopathy and well preserved vision of trial participants such that the trial was likely underpowered to demonstrate any benefit on these outcomes.

Accumulating evidence suggests that fenofibrate mediates its effect directly within the eye rather than through a reduction in circulating atherogenic lipids. In LENS, absolute reductions in atherogenic lipids were small (similar to ACCORD Lipid^17^). Studies conducted in animal models of diabetic retinopathy have shown that both oral administration and intra-vitreal injection of fenofibrate reduce retinal vascular leakage and retinal inflammation, while a study of human retinal pigment epithelium cells suggests that fenofibric acid may play a protective role on the blood-retina barrier.^18,19^

The trial addresses a major cause of visual loss^1^ for which there are limited therapeutic options. Other key strengths of the trial include its positioning within routine healthcare, allowing us to recruit almost 10% of potentially eligible individuals across mainland Scotland and follow them in a streamlined fashion. Trial participants were similar to the potentially eligible population with regard to key risk factors for diabetic retinopathy progression^9^ and the design of the trial allowed it to continue largely unaffected by the COVID-19 pandemic, apart from temporary cessation of the national DES programme in April 2020. Linkage to the national DES programme has also provided a biobank of approximately 9000 retinal images to facilitate further research. Adherence to assigned trial treatment was excellent and completeness of follow up was almost 100%. There are important limitations however. Participants with impaired kidney function needed to take trial treatment on alternate days due to the lack of a lower dose tablet, though we noted no difference in the proportional effect on the primary outcome in this group. Retinal screening in Scotland is similar to other well-established programmes using a grading scheme aligned to the ETDRS classification used in research studies^20^ with a stepwise severity scale where worse grades are increasingly predictive of developing referable disease^21^ and eligibility criteria mapped closely to ETDRS levels 20/35. However the NHS Scotland scheme does not distinguish retinopathy with the same granularity as ETDRS and it was not possible to map between the two grading schemes. Single field macula-centered 45-degree images cover a restricted retinal area (compared to ETDRS 7-standard field 30-degree imaging), excluding some of the peripheral retina (though it includes the area of the retina most likely to cause vision-threatening disease and is unlikely that the effect of fenofibrate therapy should differ across imaged and unimaged areas of the retina). Mydriasis was not used during the majority of retinal screening episodes, but 95% of images still had a high quality rating. The primary outcome included development of referable maculopathy, a grading that demonstrates disease progression and prompts further investigation for macular edema and subsequent surveillance, but which often does not require treatment in the short term. However, the reduction in referable maculopathy we observed was supported by a reduction in macular edema, and it is also quantitatively similar to results from the FIELD trial for laser treatment of macular edema, supporting its inclusion in the primary outcome.^6^ Optical coherence tomography (OCT) imaging is not performed routinely during retinal screening and we did not seek information regarding OCT that might have occurred during outpatient ophthalmology visits that were not arranged by the DES program or did not include treatment for retinopathy or maculopathy, so it is possible that some cases of macular edema were missed. However it is unlikely that this could have occurred frequently without subsequent detection of referable disease by routine retinal screening or by the need for treatment at a later stage. Adherence to trial treatment was self-reported, but results were supported by the reductions in triglycerides and eGFR, both expected effects of fenofibrate therapy. People were invited without knowledge of their ethnicity but the trial included few non-Caucasian participants.

In summary, among participants with early diabetic retinal changes, fenofibrate treatment led to a reduction in the progression of diabetic retinopathy compared to placebo.

## Supplementary Material

Supplement

## Figures and Tables

**Table 1:** Characteristics of LENS patients at baseline^*^

	Fenofibrate (N=576)	Placebo (N=575)
Age - yr	60.8 ± 12.4	60.6 ± 12.3
Female sex - no. (%)	156 (27%)	156 (27%)
Race – no. (%)^†^		
White	567 (98%)	558 (97%)
Other	9 (2%)	17 (3%)
Type of diabetes mellitus - no. (%)		
Type 1	154 (27%)	151 (26%)
Type 2	421 (73%)	423 (74%)
Other	1 (0%)	1 (0%)
Duration of diabetes – yr	18.3 ± 10.5	17.7 ± 10.0
Retinopathy grading (worse eye) – no. (%)^‡^		
No retinopathy (R0)	5 (1%)	4 (1%)
Mild background retinopathy (R1)	562 (98%)	564 (98%)
Observable background retinopathy (R2)	9 (2%)	7 (1%)
Maculopathy grading (worse eye) – no. (%)^‡^		
No maculopathy (M0)	517 (90%)	515 (90%)
Observable diabetic maculopathy (M1)	59 (10%)	60 (10%)
Retinal laser treatment or intravitreal injections or vitrectomy	53 (9%)	59 (10%)
− no. (%)		
Cardiovascular disease – no. (%)^§^	103 (18%)	96 (17%)
Blood pressure – mmHg^‖¶^		
Systolic	136.7 ± 17.2	136.4 ± 17.9
Diastolic	75.4 ± 9.4	75.5 ± 9.3
Body mass index – kg/m^2^^‖¶**^	30.9 ± 6.4	30.7 ± 6.0
HbA1c – mmol/mol^¶††^	66 ± 16	66 ± 16
Creatinine (mg/dL)		
Screening	0.88 ± 0.21	0.88 ± 0.21
Randomization	1.01 ± 0.26	1.00 ± 0.25
Estimated GFR		
Screening – no. (%)		
<60 mL/min/1.73m^2^	58 (10%)	40 (7%)
≥60 mL/min/1.73m^2^	518 (90%)	535 (93%)
Randomization – no. (%)		
<60 mL/min/1.73m^2^	130 (23%)	131 (23%)
≥60 mL/min/1.73m^2^	446 (77%)	444 (77%)
Total cholesterol – mg/dL^¶††^	156 ± 37	157 ± 38
HDL cholesterol – mg/dL^¶††^	51 ± 16	50 ± 15
Triglycerides (IQR) – mg/dL^¶††^	137 (90-205)	138 (97-195)
Baseline medications – no. (%)		
Non-insulin glucose-lowering therapy	396 (69%)	389 (68%)
Insulin	256 (44%)	249 (43%)
Statin	425 (74%)	429 (75%)
Renin–angiotensin system inhibitor	345 (60%)	341 (59%)

* Plus–minus values are means ± SD, triglycerides are shown as median (interquartile range). Percentages may not total 100 because of rounding. IQR denotes interquartile range. To convert the values for cholesterol to millimoles per liter multiply by 0.02586. To convert the values for triglycerides to millimoles per liter multiply by 0.01129.

† Race was reported by the participants.

‡ Retinopathy and maculopathy gradings were recorded from the SCI Diabetes system up to randomization; worse eye retinopathy was defined as R2 > R1 > R0; worse eye maculopathy defined as M1 > M0.

§ Cardiovascular disease was defined as previous myocardial infarction, coronary revascularization, non-coronary revascularization, stroke or transient ischemic attack.

‖ Based on measurements at the randomization assessment.

¶ Missing data: blood pressure 70 participants, BMI 35 participants, HbA1c 74 participants, total cholesterol 12 participants, HDL cholesterol 23 participants, triglycerides 17 participants.

** The body-mass index is the weight in kilograms divided by the square of the height in meters.

†† Based on blood tests at the screening assessment.

**Table 2:** Effect on renal function, lipids, HbA1c and urine albumin creatinine ratio^*^

	Fenofibrate		Placebo		
	N	Mean (95% CI)	N	Mean (95% CI)	Difference between groups (95% CI)
eGFR – mL/min/1.73m^2^					
Baseline	576	86.9 (85.4-88.4)	575	87.2 (85.7-88.7)	
Trial-average follow-up^†^		73.9 (73.1-74.7)		81.9 (81.1-82.7)	-7.9 (-9.1 - -6.8)
Total cholesterol – mg/dL					
Baseline	572	156.4 (153.4-159.4)	567	156.8 (153.6-159.9)	
Trial-average follow-up^†^		152.3 (150.4-154.3)		156.5 (154.6-158.5)	-4.2 (-7.0 - -1.4)
Non-HDL cholesterol – mg/dL					
Baseline	568	105.8 (103.0-108.7)	560	106.2 (103.1-109.3)	
Trial-average follow-up^†^		103.3 (101.3-105.3)		106.3 (104.3-108.3)	-3.0 (-5.8 - -0.2)
HDL cholesterol – mg/dL					
Baseline	568	50.6 (49.2-51.9)	560	50.4 (49.2-51.6)	
Trial-average follow-up^†^		48.6 (48.0-49.2)		49.9 (49.3-50.5)	-1.2 (-2.1 - -0.4)
Triglycerides – mg/dL					
Baseline	571	140.2 (133.5-147.2)	563	136.7 (130.7-143.0)	
Trial-average follow-up^†^		118.0 (113.7-122.4)		136.7 (131.7-142.0)	-13.7% (-18.1% - -9.1%)
HbA1c – mmol/mol					
Baseline	538	66.4 (65.0-67.7)	539	66.3 (65.0-67.7)	
Trial-average follow-up^†^		66.7 (65.8-67.6)		66.8 (65.9-67.8)	-0.2 (-1.5 - 1.2)
					
Urine albumin creatinine ratio – mg/g					
Baseline	312	14.4 (12.3-16.9)	310	16.6 (14.0-19.6)	
Trial-average follow-up1^†^		13.6 (12.1-15.3)		15.5 (13.8-17.5)	-12.4% (-25.8% - 3.5%)

* Shown are arithmetic means and confidence intervals for eGFR, total cholesterol, non-HDL cholesterol, HDL cholesterol and HbA1c; shown are geometric means and approximate 95% confidence intervals for triglycerides and urine albumin to creatinine ratio. Confidence intervals have not been adjusted for multiplicity, and should not be used to infer clinical utility. For triglycerides and the urine albumin to creatinine ratio, the difference between groups reflects a percentage difference. To convert the values for cholesterol to millimoles per liter multiply by 0.02586. To convert the values for triglycerides to millimoles per liter multiply by 0.01129.

† The estimates were derived from a linear mixed model repeated measures adjusted for the baseline value and the minimization criteria.
