# Supplementary material for: Effect of Fenofibrate on Progression of Diabetic Retinopathy
Source: NEJM Evid. Author manuscript; Available in PMC 2024 Jul 24. (PMC7616293; doi:10.1056/EVIDoa2400179)
Supplement: Supplement [file EMS197105-supplement-Supplement.pdf]

This appendix has been provided by the authors to give readers additional information about their work.

SUPPLEMENTARY APPENDIX TO:

Effect of Fenofibrate on Progression of Diabetic Retinopathy

David Preiss, Ph.D., F.R.C.Path., M.R.C.P., Jennifer Logue, M.D., F.R.C.Path., M.R.C.P., Emily Sammons, D.Phil., et al for the LENS Collaborative Group

Table of contents

Members of the LENS Collaborative Group 3
Roles of Members of the LENS Collaborative Group 5
Information regarding Scotland's Diabetic Eye Screening programme 9
Adjudication Charter and Outcome Definitions 11
Trial treatment mailing error 26
Clarification regarding primary outcome terminology 27
Figure S1. CONSORT diagram of the LENS trial 28
Figure S2. Effect of fenofibrate compared to placebo on eGFR 29
Table S1. NHS Scotland's Diabetic Eye Screening Programme grading scheme for retinopathy and maculopathy 30
Table S2. Serious Adverse Events in run-in, categorized by MedDRA system organ class 31
Table S3. Representativeness of the trial population 32
Table S4. Completeness of Follow up 33
Table S5. Frequency of retinal screening after randomization 34
Table S6. Modalities of retinal screening and information on retinal image quality and the use of mydriasis 35
Table S7. Adherence to randomized trial treatment 36
Table S8. Reasons for stopping randomized trial treatment 37
Table S9. Effect of fenofibrate compared to placebo on treatment for diabetic retinopathy or maculopathy 39
Table S10. Effect of fenofibrate compared to placebo on visual acuity, quality of life and visual function* 40
Table S11. Effect of fenofibrate compared to placebo on major cardiovascular events and non-traumatic lower limb amputations 42
Table S12. Effect of fenofibrate compared to placebo on renal function, lipids, HbA1c and urine albumin creatinine ratio by year and as trial-average* 43
Table S13. Serious adverse events after randomization, categorized by MedDRA system organ class 47

Members of the LENS Collaborative Group

Members of the LENS Collaborative Group include colleagues listed below and the manuscript authors (i.e. Writing Committee members).

Paula Williamson, Jonathan Bodansky, Allan Cairns, Sue Dickie, Gillian Hallard, Gozie Joe Adigwe, Laura Jones, Timothy Lyons, Isla Mackenzie, Dermot Neely, Sarah Wild, Ian Young, Praveen Patel, Alan Watkins, Natalie Staplin, Sarah Howard, Kate Sawyer, Karen Taylor, Patricia Achiri, Andy Burke, Susan Hurley, Gareth McChlery, Kevin Murphy, Sandra Pickworth, Alison Timadjer, Kate Gardiner, Monique Willett, Liz Wincott, Matthew Beesley, Charles Cutting, Monika Raj, Ian Roure, Michelle Goonasekera, Graham Bagley, Anne Whitehouse, Isobel Young, Gavin Brown, Kate Bird, Heather Halls, Guanguo Cui, Charles Daniels, Angela Field, Bob Goodenough, Youcef Mostefai, Samee Syed, Angela Chamberlain, Carol Knott, Anna Liew, Juliette Meek, Meera Mistry, Andrea Wilson, Patrick Zettegren, Rach AitSadi, Ian Barton, Alex Baxter, Clive Berry, Rijo Kurien, Michael Lay, Aleksandra Murawska, James Thompson, Allen Young, Enti Spata, Rachel Raff, Crispian Howard, Diann Taggart, Paul Connelly, Mohan Varikkara, Scott Blackwell, Fiona Elliott, Jacqueline Locke, Angela Sweeney, Stephen Wood, Olive Herlihy, Jeni Darling, Jill Little, Ewan Bell, Susan Boytha, Paula Neill, Caroline Styles, Andrew Blaikie, Laura Beveridge, Tina Coventry, Susan Fowler, Karen Gray, Linda Lister, Amanda McGregor, Sandra Pirie, Evgeniya Postovalova, Anna White, John Doig, Stephanie Brogan, Margie Duncan, Angela Gilmour, Lynn Ryan, Anne Todd, Lesley Whitelaw, Elaine Wilson, Sam Philip, Ann Cadzow, Sharlene Greig, Siby Joseph, Lynne Murray, Julie Toye, Sonia Zachariah, David Carty, Lindsey Bailey, Alison Begg, Katherine Brown, Charlotte Clark, Katharine Duffy, Shirley Fawcett, Adam Gill, Emma Hughes, David Jenkins, Laila Martin, Christine McAllister, Mhairi McIntyre, Leeanne Milne, Erin Murphy, Hilary Peddie, Katharine Sharp, Susan Speirs, Joyce Thompson, Lynne Turner, Simon

Hewick, Clare Bradley, Jim Finlayson, Laura O'Keeffe, Meena Virdi, Soo Park, Murdina Bell, Maureen Brown, Linda Macliver, Jackie Quigley, Karen Madill, Daniel Beck, Freddie Burgess, Karen Charlwood, Lauren Finlayson, Nicola Freeman, Margaret McDonald, Carla Pettifor, Karen Sedstrem, Alice Thomson, Ellen Malcolm, Jackie Lindsay, Lisa Stewart, Helen Colhoun, Robert Lindsay, John Olson, Naveed Sattar, Graham Scotland

Roles of Members of the LENS Collaborative Group

Members are based in the United Kingdom unless otherwise indicated.

Writing Committee: David Preiss, Jennifer Logue, Emily Sammons, Mohammed Zayed, Jonathan Emberson, Rachel Wade, Karl Wallendszus, Will Stevens, Rosanna Cretney, Simon Harding, Graham Leese, Gemma Currie, Jane Armitage

Steering Committee: Paula Williamson (Chairperson, University of Liverpool)*, David Preiss (Chief Investigator, University of Oxford), Jane Armitage (University of Oxford), Jonathan Bodansky (Leeds Teaching Hospitals NHS Trust)*, Allan Cairns (patient representative)*, Sue Dickie (patient representative)*, Jonathan Emberson (University of Oxford), Gillian Hallard (Royal National Institute of Blind People (RNIB) Scotland)*, Simon Harding (University of Liverpool)*, Gozie Joe Adigwe (previous member, RNIB Scotland)*, Laura Jones (previous member, RNIB Scotland)*, Jennifer Logue (University of Lancaster), Timothy Lyons (Medical University of South Carolina, USA)*, Isla Mackenzie (University of Dundee), Dermot Neely (Newcastle upon Tyne Hospitals NHS Foundation Trust)*, Sarah Wild (University of Edinburgh) [*independent members]

Data Monitoring Committee: Ian Young (Chairperson, Queen's University Belfast), Praveen Patel (Moorfields Eye Hospital), Alan Watkins (University of Swansea), Natalie Staplin (Non-voting statistician, University of Oxford)

Central Coordinating Office (University of Oxford):

Central Coordinating Office Management Team: Jane Armitage, Sarah Howard, David Preiss, Emily Sammons, Kate Sawyer, Karen Taylor, Mohammed Zayed; *Project Management*: Sarah Howard, Kate Sawyer, Karen Taylor; *Administration*: Patricia

Achiri, Andy Burke, Susan Hurley, Gareth McChlery, Kevin Murphy, Sandra Pickworth, Alison Timadjer, Kate Gardiner, Monique Willett, Liz Wincott; *Call centre*: Matthew Beesley, Charles Cutting, Monika Raj, Ian Roure; *Clinical*: David Preiss, Emily Sammons, Mohammed Zayed, Michelle Goonasekera; *Communications*: Graham Bagley, Anne Whitehouse, Isobel Young; *Contracts*: Gavin Brown; *Data analysis*: Kate Bird, Rosanna Cretney, Heather Halls, Will Stevens, Karl Wallendszus; *IT Validation*: Guanguo Cui, Charles Daniels, Angela Field, Bob Goodenough, Youcef Mostefai, Samee Syed; *Monitoring*: Angela Chamberlain, Carol Knott, Anna Liew, Juliette Meek, Meera Mistry, Andrea Wilson, Patrick Zettegren; *Programming*: Rach AitSadi, Ian Barton, Alex Baxter, Clive Berry, Rosanna Cretney, Rijo Kurien, Michael Lay, Aleksandra Murawska, James Thompson, Allen Young; *Statistics*: Jonathan Emberson, Enti Spata, Rachel Wade; *Systems Analyst*: Rachel Raff; *Transport*: Crispian Howard

Regional Centre Team (NHS Greater Glasgow and Clyde):

Project Management: Diann Taggart; *Clinical*: Gemma Currie, Paul Connelly, Jennifer Logue

Local Clinical Centres:

NHS Ayrshire and Arran (University Hospital Ayr, University Hospital Crosshouse; 106 participants randomized): Mohan Varikkara (Lead Investigator), Scott Blackwell, Fiona Elliott, Jacqueline Locke, Angela Sweeney, Stephen Wood; *NHS Borders (Borders General Hospital; 42 participants randomized)*: Olive Herlihy (Lead Investigator), Jeni Darling, Jill Little; *NHS Dumfries and Galloway (Dumfries and Galloway Royal Infirmary; 34 participants randomized)*: Ewan Bell (Lead Investigator), Susan Boytha, Paula Neill; *NHS Fife (Queen Margaret Hospital, Victoria Hospital*; *195 participants randomized*): Caroline Styles (Lead Investigator), Andrew Blaikie, Laura Beveridge,

Tina Coventry, Susan Fowler, Karen Gray, Linda Lister, Amanda McGregor, Sandra Pirie, Evgeniya Postovalova*; NHS Forth Valley (Forth Valley Royal Hospital; 97 participants randomized)*: Anna White (Lead Investigator), John Doig (previous Lead Investigator), Stephanie Brogan, Margie Duncan, Angela Gilmour, Lynn Ryan, Anne Todd, Lesley Whitelaw, Elaine Wilson; *NHS Grampian (Aberdeen Royal Infirmary; 93 participants randomized)*: Sam Philip (Lead Investigator), Ann Cadzow, Sharlene Greig, Siby Joseph, Lynne Murray, Julie Toye; *NHS Greater Glasgow and Clyde (Glasgow Royal Infirmary, Queen Elizabeth University Hospital; 108 participants randomized)*: Sonia Zachariah (Local Lead Investigator (joint)), David Carty (Local Lead Investigator (joint)), Lindsey Bailey, Alison Begg, Katherine Brown, Charlotte Clark, Katharine Duffy, Shirley Fawcett, Adam Gill, Emma Hughes, David Jenkins, Laila Martin, Christine McAllister, Mhairi McIntyre, Leeanne Milne, Erin Murphy, Hilary Peddie, Katharine Sharp, Susan Speirs, Joyce Thompson, Lynne Turner; *NHS Highland (Raigmore Hospital; 45 participants randomized)*: Simon Hewick (Local Lead Investigator), Clare Bradley, Jim Finlayson, Laura O'Keeffe; *NHS Lanarkshire (University Hospital Hairmyers, University Hospital Monklands, University Hospital Wishaw; 204 participants randomized)*: Meena Virdi (Local Lead Investigator), Soo Park, Murdina Bell, Maureen Brown, Linda Macliver, Jackie Quigley; *NHS Lothian (Princess Alexandra Eye Pavilion; 80 participants randomized)*: Karen Madill (Local Lead Investigator), Daniel Beck, Freddie Burgess, Karen Charlwood, Lauren Finlayson, Nicola Freeman, Margaret McDonald, Carla Pettifor, Karen Sedstrem, Alice Thomson; *NHS Tayside (Ninewells Hospital; 147 participants randomized)*: Graham Leese (Local Lead Investigator), Ellen Malcolm, Jackie Lindsay, Lisa Stewart

LENS Collaborating Investigators: David Preiss (University of Oxford), Jane Armitage (University of Oxford), Helen Colhoun (University of Edinburgh), Gemma Currie (University of Glasgow), Graham Leese (University of Dundee), Robert Lindsay (University of Glasgow), Jennifer Logue (University of Lancaster), John Olson (NHS

Grampian), Naveed Sattar (University of Glasgow), Emily Sammons (University of Oxford), Graham Scotland (University of Aberdeen)

Information regarding Scotland's Diabetic Eye Screening programme

The National Health Service in Scotland, United Kingdom, offers population screening to adults for abdominal aortic aneurysms, bowel cancer, breast cancer, cervical cancer, and diabetic retinopathy. The Diabetic Eye Screening (DES) programme was initiated in 2003. Diabetic eye screening (i.e. retinal screening) is offered to people aged 12 and over with diabetes. The frequency of invitation is based on an individual's recent retinal screening results (see Table S1). At each retinal screening visit, a single 45-degree macula-centered color photograph of each eye is captured by a trained imager with a non-mydriatic camera through an undilated pupil. If the imager considers an image to be of insufficient quality for grading, they then apply mydriatic drops and capture additional images. If it is still not possible to capture gradable images, then the individual is referred for slit lamp examination (and may remain under slit lamp review within the DES programme in the longer term). Image quality is graded (from best to worst) as - nerve fibre layer visible, nerve fibre layer not visible, small vessels blurred, major arcade vessels just blurred, image not gradable/technical failure.

Retinal images are graded by trained professionals in ten centres according to the NHS Scotland Diabetic Eye Screening scheme (see Table S1). Images undergo three levels of grading - Level 1 grading (typically conducted using image analysis software) to identify images with retinal disease, Level 2 grading (by junior graders) to identify images with potentially sight-threatening disease and Level 3 (by senior graders) to make the final grading decision regarding which patients require specialist referral or further investigation. Graders participate in an external quality assurance exercise in both Spring and Fall annually. Individuals with R3 (referable background retinopathy i.e. moderately severe or severe non-proliferative retinopathy) or R4 (proliferative retinopathy) disease are referred for specialist ophthalmological review. Until approximately early 2022, all individuals with M2 (referable maculopathy i.e. blot

hemorrhage or exudate within 1 disc diameter distance of the foveal centre) disease were also referred for specialist review. Since then there has been a phased introduction of a new management pathway whereby those with M2 disease and good visual acuity remain within the screening programme (and are reviewed every six months) whereas those with M2 disease and poor visual acuity (6/9.5 or worse) have optical coherence tomography (OCT) imaging performed every six months followed by specialist ophthalmological referral if there is evidence of significant central macular edema.

A randomised placebo-controlled trial of fenofibrate to prevent progression of non-proliferative retinopathy in diabetes.

Adjudication Charter and Outcome Definitions

EDMS #6781
Version 1.3

Version History

**Version**	**Date**	**Summary**<br>First draft version	**Author**
0.1	9th May 2020		David Preiss
1.0	10th June 2020	Final version	David Preiss
1.1	29th April 2021	Minor edits	David Preiss
1.2	29th June 2022	Minor edits	David Preiss
1.3	10th January 2023	Add adjudication statuses; clarify which events are adjudicated	David Preiss

CONTENTS

1 Overview 14
2 Verification of events 14
3 Adjudication of events 15
3.1 ROLES 15
3.2 RESPONSIBILITIES 15
3.3 METHODS 16
3.3.1 General approach to adjudication *16*
3.3.2 Sources of information *16*
3.3.3 Invalid and duplicate events *17*
3.3.4 Handling of dates *17*
3.3.5 Data items available for adjudication *17*
3.3.6 Adequacy of information *19*
3.4 IT AND CODING SYSTEMS 20
4 Study Outcomes and Definitions 20
4.1 LENS TRIAL OUTCOMES 21
4.1.1 Primary outcome *21*
4.1.2 Secondary outcomes *21*
4.1.3 Tertiary outcomes *21*
4.2 DEFINITIONS OF OUTCOMES 22
4.2.1 Progression from observable DR to referable DR *22*
4.2.2 Treatment for DR *22*
4.2.3 Any progression of DR across the DES grading scale *23*
4.2.4 Visual acuity *23*
4.2.5 The development of hard exudates or blot haemorrhage within 1 disc diameter of the foveal centre *23*
4.2.6 The development of macular oedema *23*
4.2.7 Visual function *24*
4.2.8 Quality of life *24*
4.2.9 Urine albumin: creatinine ratio *24*
4.2.10 Major cardiovascular events *24*
4.2.11 Lower limb amputation *24*
5 Appendix 1: NHS Scotland DES Collaborative grading system 25

1. Overview

The purpose of this document is to provide definitions of primary, secondary and tertiary outcomes for the LENS trial, and to outline the roles, responsibilities and procedures in regards to the verification and adjudication of these outcomes.

There are two main methods to identify clinical outcomes such as eye events and cardiovascular outcomes, namely 6-monthly telephone follow-up assessments with participants during which they are asked whether events of interest have occurred since the previous contact (in which case an adverse event [AE] is recorded), medical records follow-up where a participant cannot be contacted or has withdrawn consent for direct contact, and also linkage to NHS Scotland datasets.

LENS will use two complementary approaches to handle potential clinical outcomes: (i) verification (ii) adjudication. Verification is the process of establishing the validity of an adverse event report, whereas adjudication is the formal process of phenotyping adverse events from supporting documentation, against a pre-defined standard. A distinction is made with regards to the handling of events which are to be verified and those which are to be adjudicated (see Sections 2 and 3).

As stated in the protocol, ‘diabetic retinopathy’ (DR) should be considered to refer to both diabetic retinopathy and diabetic maculopathy unless otherwise specified.

2. Verification of events

The following types of events will undergo verification in the web-based management system, *Endeavour:*

- cardiovascular events
  - myocardial infarction
  - stroke
  - coronary artery revascularizations
  - peripheral artery revascularizations
- lower limb amputations
- deaths

When one of these event types is first reported, it is given a status of “unconfirmed.” Clinical users from the Local Clinical Centre [LCC] or Central Coordinating Office [CCO] will then use information available to them (e.g. medical records) to assess whether the reported AE occurred. Unlike adjudicated events, LCCs will not be expected to send any documentation about verified AEs to the CCO (though this can be done where assistance is

sought by the LCC). Users will review verification events based on the available information, and assign the following general statuses to them:

- ‘Confirmed in the medical notes’
- ‘Unable to confirm in the medical notes’
- Refuted

The motivation to verify, but not adjudicate, these clinical AEs is supported by the fact that large cardiovascular trials (FIELD and ACCORD-Lipid) have already provided detailed evaluations (with adjudication of cardiovascular events and deaths) of the effect of fenofibrate therapy on such outcomes.

3. Adjudication of events

According to the LENS protocol, data regarding the progression to referable eye disease shall mostly be obtained through linkage to the NHS Scotland registries, however a small number of additional events are to be adjudicated. These include:

- Eye events reported as AEs:
    - Medical eye procedures for DR:
        - Laser treatment (i.e. photocoagulation)
        - Intra-vitreal injection
        - Vitrectomy
    - Vitreous haemorrhage
    - Macular oedema
- SSARs[1]

3.1 Roles

Event adjudication is to be carried out by medically qualified staff working within the University of Oxford’s Clinical Trial Service Unit. Adjudication staff are to be given appropriate training, covering relevant aspects of the Trial Protocol, the procedures and IT systems relating to data capture and adjudication, and the specific issues relating to those aspects of the study which they are adjudicating.

3.2 Responsibilities

Adjudication staff are responsible for:

- Reading and understanding the contents of the LENS protocol, relevant SOPs, and the Outcome Definitions and Adjudication Charter
- Thorough review of all supporting documentation (henceforth referred to as “Event Data Packages”) after receipt of complete packages at the CCO
- Completion of the adjudication process within the *Endeavour* system

[1] Handling of SSARs is discussed in EDMS #5441 *Reporting of Adverse Events and Procedures for Un-blinding Treatment Allocation for the LENS Trial*

- Escalating any difficult adjudication cases to a senior adjudicator (see section 3.3.1)

3.3 Methods

3.3.1 General approach to adjudication

All Event Data Packages will be redacted at the LCC to remove references to identifiers and results of lipid blood tests (though it is unexpected for lipid blood results to be included in ophthalmology letters and reports). The unique LENS participant ID and AE ID should be added to each page of the Event Data Package. All adjudicators will be blinded to randomized treatment allocation. Only one adjudicator will execute the adjudication procedure for each event. Where assistance is required, the adjudicator will be able to discuss the event with a senior adjudicator to reach resolution on the event by consensus. Where the adjudicator considers there to be any risk of unblinding based on the information provided, the adjudicator will cease adjudication of that event, liaise with the LCC to provide appropriate documentation (e.g. with additional redaction) and will then transfer responsibility for adjudicating the relevant event to an alternative adjudicator.

This approach to adjudication has been applied in various cardiovascular trials in CTSU to good effect.

Definitions of the outcomes to be adjudicated are given in Section 4. In all cases, clinical judgement should be used; some cases may be complex and may not neatly fit the criteria set out. Furthermore, within a large-scale multicentre national study, some results may not be available, either because they were never measured by the physician responsible for the participant's care at the time, or because the test was not available locally, or because the results can no longer be found. In such cases, the most appropriate preferred term (based on the currently available information) should be selected.

3.3.2 Sources of information

When adjudicating potential outcome measures, adjudicators should take into account all available sources of information. These may include:

- Study Forms: including study visits and AE reports in *Endeavour*
- Supporting Documentation: documentation provided to the CCO by the LCCs as part of the adjudication process
- Routine NHS data sources: information available from NHS Scotland registries, where available (in particular NHS Scotland Diabetic Eye Screening [DES] program data)
- Study 'Contact Log': the Contact Log section of *Endeavour* contains correspondence relating to the study participants

3.3.3 Invalid and duplicate events

An AE report should only be judged to be invalid if the event never occurred for this participant or if it does not meet the definition of an AE. *Endeavour* allows AEs to be marked as invalid where required. Duplicate reports of the same event may be identified during the adjudication process and the duplicate(s) will be marked as invalid.

3.3.4 Handling of dates

For those events that require adjudication, the event start date is to be checked and amended in line with the available supporting documentation. If the date of event is unclear, additional information need only be sought if it would be likely to determine the pre- / post-randomization status.

The event date must never be after the date that the AE was initially reported and this will be enforced by an automated check within the *Endeavour* system.

Particular care should be taken if editing the event date would change the randomization status at the time of the event (i.e. moving the date from after to before the date of randomization or vice versa).

3.3.5 Data items available for adjudication

The following aspects of any AE report should be reviewed (and edited where appropriate) as part of the adjudication process:

Field	Options
Source of event	• Participant<br>• Relative / friend<br>• Study nurse<br>• Study doctor<br>• Other doctor<br>• Other nurse<br>• Medical records<br>• Data linkage
Valid	• Yes<br>• No
Description	MedDRA preferred term (or extra term)
Start date	Event start date
Death date*	Date of death (fatal events only)
End date	Date event ended (if recovered)

Field	Options
Serious	• Yes<br>• No
Reason for seriousness†	• Death* (fatal events only)<br>• Life-threatening<br>• Hospitalization<br>• Disabling<br>• Congenital anomaly in offspring<br>• Other important medical condition
Location†	• Place: freetext<br>• Town/city: freetext<br>• Name of doctor: freetext
Nights in hospital†	Number of nights in hospital
Related	• Yes<br>• No
Outcome	• Recovered<br>• Recovering<br>• Not recovered<br>• Death* (fatal outcomes only)<br>• Unknown
Adjudication status‡	• Needs documentation to be sent<br>• Documentation has been sent (1)<br>• Documentation received at CCO (1)<br>• Confirmed in medical notes as entered (2)<br>• Confirmed in medical notes, edited (2)<br>• Unable to confirm in medical notes (2)<br>• Refuted (2)<br>• Confirmed in medical notes as entered, retinopathy (2)<br>• Confirmed in medical notes as entered, maculopathy (2)<br>• Confirmed in medical notes as entered, not due to diabetes (2)

* only relevant for fatal SAEs
† only relevant for SAEs
‡ statuses and transitions are described in section 3.3.6.2; (1) indicates statuses relevant to administrative tasks, (2) indicates statuses set by adjudicators

3.3.6 Adequacy of information

3.3.6.1 General rules

For an Eye event to be considered as fully assessed, the following data items must be confirmed:

- Event start date (in particular, whether pre-/post-randomization)
- Event description (preferred term or extra term code)
- Adjudication status (confirmed, unable to confirm, refuted)

3.3.6.2 Recording Adjudication status

Adjudication Status is stored in the study database for relevant reported AEs as follows:

Adjudication Status	Status Set by	Description	Action
Needs documentation to be sent	Initial value set by *Endeavour*	No adjudication conducted.	LCC to send documents
Documentation has been sent*	LCC user or CCO user	Package sent to CCO	CCO to confirm receipt
Documentation received at CCO*	CCO user	Package received at CCO	Ready to adjudicate
Confirmed in medical notes as entered†¶	Adjudicator	Adjudicator confirmed event occurred as entered; for retinal laser therapy and intra-vitreal injection AEs, this status will be used when treatment was required for diabetic retinopathy but it is unclear whether this was primarily for retinal disease or macular disease	Adjudicated, no further action
Confirmed in medical notes, edited†¶	Adjudicator	Adjudicator confirms an eye event occurred; preferred term edited to other eye event	Adjudicated, no further action
Unable to confirm in medical notes†¶	Adjudicator	This remains the preferred term for the event; all potential sources of information have been exhausted without being able to confirm or refute key data items for the event	None; or further data sought

Refuted[†]	Adjudicator	Available information confirms that the reported event did not occur	None
Confirmed in medical notes as entered, retinopathy[†¶]	Adjudicator	Adjudicator confirmed event occurred as entered; this status will be used for retinal laser therapy and intra-vitreal injection AEs, when treatment was required for retinal (not macular) disease	Adjudicated, no further action
Confirmed in medical notes as entered, maculopathy[†¶]	Adjudicator	Adjudicator confirmed event occurred as entered; this status will be used for retinal laser therapy and intra-vitreal injection AEs, when treatment was required for macular (not retinal) disease	Adjudicated, no further action
Confirmed in medical notes as entered, not due to diabetes[†§]	Adjudicator	Adjudicator confirmed event occurred as entered; however, underlying cause of the condition was not diabetic retinopathy	Adjudicated, no further action

* administrative tasks
† adjudication tasks
¶ AEs adjudicated to these statuses will count towards pre-specified eye outcomes
§ AEs adjudicated to this status will not count towards pre-specified eye outcomes when adjudged to not be caused by diabetic retinopathy according to the best interpretation of the adjudicator (but will be reported elsewhere [e.g. results table footnote])

3.4 IT and coding systems

All adjudication will be performed using the AE module of the LENS web-based IT system *Endeavour*.

All event descriptions are to be coded using preferred terms from MedDRA version 14.0 (to which a small number of additional terms relevant to the trial have been added). Adjudicators should use the available supporting information to choose the most suitable preferred term available.

4. Study Outcomes and Definitions

This section describes LENS trial outcomes and the definitions of these outcomes. With the exception of health economic analyses, these definitions were finalised (in version 1.0 of this document) prior to review of any

unblinded Eye event data by the Data Monitoring Committee and prior to receipt of any NHS linkage data.

The approach to statistical analyses of these outcomes will be described separately in the LENS Statistical Analysis Plan and is not considered here.

4.1 LENS trial outcomes

Pre-specified outcomes, as listed in the LENS protocol, are provided below.

4.1.1 Primary outcome

The composite of:

- Progression from observable DR to referable DR, or
- Treatment for DR with:
  - Retinal laser therapy
  - Vitrectomy
  - Intra-vitreal injection of medication

4.1.2 Secondary outcomes

- The individual components of the composite primary outcome, i.e.:
  - Progression of DR to referable DR
  - Retinal laser therapy for DR
  - Vitrectomy for DR
  - Intra-vitreal injection for DR
- Composite of treatments (i.e. retinal laser, vitrectomy or intra-vitreal injection) for DR
- Any progression of DR across the DES grading scale
- Visual acuity
- The development of hard exudates or blot haemorrhage within 1 disc diameter of the macula
- The development of macular oedema
- Visual function
- Quality of life
- Total cost to the health service (further detail not included here)
- Cost-effectiveness (further detail not included here)

4.1.3 Tertiary outcomes

- Urine albumin: creatinine ratio
- Composite of major cardiovascular events (myocardial infarction, stroke, coronary artery revascularization and peripheral artery revascularisation)
- Composite of non traumatic lower limb amputation (minor and major)

4.2 Definitions of Outcomes

4.2.1 Progression from observable DR to referable DR

It is expected that the vast majority of progression to referable DR outcomes will be identified by linkage to NHS Scotland registries. In addition, referable disease identified during clinical examination, namely vitreous haemorrhage, macular oedema, pre-proliferative and/or proliferative retinopathy, will be recorded as AEs and will also be counted towards relevant outcomes (after adjudication where required). Relevant outcomes will be reported in terms of numbers of participants, not individual eyes.

Referable retinopathy or maculopathy from NHS data linkage: referable DR in the context of the LENS trial is defined according to the NHS Scotland retinal screening grading scheme as ‘R3’ or ‘R4’ or ‘M2’ in at least one eye (see Appendix 1: NHS Scotland DES Collaborative grading system). Not adjudicated.

Vitreous haemorrhage from AE report: unrefuted AE of vitreous haemorrhage (exclusion: vitreous haemorrhage not due to DR). Adjudicated.

Macular oedema from AE report: unrefuted AE of macular oedema; macular oedema refers to accumulation of fluid in the macular region with retinal thickening but does not mandate that a certain minimum threshold thickness is exceeded (exclusion: macular oedema not due to DR). Adjudicated.

Macular oedema from OCT imaging NHS Scotland DES data linkage: during LENS, NHS Scotland is introducing routine OCT imaging for patients with M2 maculopathy and poor visual acuity in a phased manner. Any report of macular oedema (namely referable central oedema, observable central oedema, observable inner ring oedema, observable outer ring oedema) will be considered as a macular oedema outcome. Not adjudicated.

Pre-proliferative retinopathy from AE report: unrefuted AE of pre-proliferative retinopathy (exclusion: pre-proliferative retinopathy not due to DR). Not adjudicated – these AEs are created by CCO clinicians during adjudication of other eye events.

Proliferative retinopathy from AE report: unrefuted AE of proliferative retinopathy (exclusion: proliferative retinopathy not due to DR). Not adjudicated – these AEs are created by CCO clinicians during adjudication of other eye events.

4.2.2 Treatment for DR

*Retinal laser therapy**: unrefuted AE of retinal laser (i.e. photocoagulation) (exclusions: laser not for retinal disease [e.g. capsular opacity, iridotomy] and retinal laser not for DR [e.g. retinal breaks, other ocular disease]). Adjudicated.

Vitrectomy: unrefuted AE of vitrectomy (exclusion: vitrectomy not for DR). Adjudicated.

*Intra-vitreal injection**: unrefuted AE of intra-vitreal injection (exclusion: intra-vitreal injection not for DR). Adjudicated.

*where possible, we will differentiate between treatments for (i) proliferative diabetic retinopathy (ii) macular oedema – see Section 3.3.6.2

4.2.3 Any progression of DR across the DES grading scale

Baseline imaging will be considered to be NHS Scotland retinal screening results from imaging conducted most recently prior to the date of randomization (see Appendix 1: NHS Scotland DES Collaborative grading system). For this outcome, only eyes recorded as R0, R1 or R2 (for retinopathy) and M0 or M1 (for maculopathy) at baseline will be considered i.e. eyes with any other baseline gradings will be excluded. This outcome will be reported in terms of numbers of participants, not individual eyes.

Progression will be considered as a composite of any ≥1 step in R or M grade as follows:

- R0 at baseline: to any of R1, R2, R3, R4, vitreous haemorrhage, macular oedema or any DR treatment
- R1 at baseline: to any of R2, R3, R4, vitreous haemorrhage, macular oedema or any DR treatment
- R2 at baseline: to any of R3, R4, vitreous haemorrhage, macular oedema or any DR treatment
- M0 at baseline: to any of M1, M2, vitreous haemorrhage, macular oedema or any DR treatment
- M1 at baseline: to any of M2, vitreous haemorrhage, macular oedema or any DR treatment

After randomization, regression followed by progression (e.g. R1 at baseline, followed by R0 and later R1 in that eye) to the initial baseline level will not be considered to represent progression unless the subsequent grading is worse than the baseline grading.

4.2.4 Visual acuity

LogMAR or Snellen measurement of visual acuity, as collected by data linkage from NHS Scotland registry data at retinal screening visits

4.2.5 The development of hard exudates or blot haemorrhage within 1 disc diameter of the foveal centre

This represents ‘M2’ in the DES grading scheme.
See Appendix 1: NHS Scotland DES Collaborative grading system.

4.2.6 The development of macular oedema

Macular oedema will consist of AEs of macular oedema plus any cases identified from NHS data linkage; see section 4.2.1

4.2.7 Visual function

Visual function will be defined by the composite score derived from the VFQ25. VFQ data are collected at the screening visit, after two years and at the end of the study.

4.2.8 Quality of life

Quality of life will be defined by EQ-5D questionnaire composite scores. EQ-5D data are collected at the screening visit, after two years and at the end of the study.

4.2.9 Urine albumin: creatinine ratio

Urine albumin: creatinine ratio (UACR) results. Urine is collected for measurement of UACR at LENS screening visits wherever possible. Thereafter, LENS does not collect further urine specimens but participants will typically have regular spot urine collections for UACR as part of routine care. Data from the baseline and routine care UACRs will be sought via linkage to NHS Scotland registries.

4.2.10 Major cardiovascular events

Cardiovascular events will be verified (not adjudicated) as described in section 2.

This outcome is a composite of myocardial infarction, stroke, coronary artery revascularisation and peripheral artery revascularisation. Unrefuted relevant AEs will be counted towards this outcome.

Myocardial infarction: AEs recorded as myocardial infarction and acute coronary syndrome (exclusion: unstable angina)

Stroke: ischaemic stroke AEs, haemorrhagic stroke AEs and stroke AEs of unclear etiology (exclusions: transient ischaemic attack, amaurosis fugax, ruptured cerebral aneurysm, subdural haematoma, subarachnoid haemorrhage)

Coronary artery revascularisation: AEs of angioplasty, stent and thrombectomy procedures of the coronary arteries; plus coronary artery bypass graft

Peripheral artery revascularisation: AEs of angioplasty, stent, endarterectomy and bypass procedures of the carotid, subclavian, iliac, limb and renal arteries, and of the aorta; plus aortic aneurysm repair

4.2.11 Lower limb amputation

This outcome is a composite of non-traumatic major and minor lower limb amputations. Unrefuted relevant AEs will be counted towards this outcome.

Minor lower limb amputation: AE of amputation distal to the ankle (i.e. foot and toe)

Major lower limb amputation: AE of amputation through or proximal to the ankle (i.e. leg)

5. Appendix 1: NHS Scotland DES Collaborative grading system

<table>
<tr><th>Grading</th><th>Description</th></tr>
<tr><td colspan="2">RETINOPATHY</td></tr>
<tr><td>R0</td><td>‘No retinopathy’</td></tr>
<tr><td>R1</td><td>‘Background DR – mild’
The presence of at least one of any of the following features anywhere
dot haemorrhages
microaneurysms
hard exudates
cotton wool spots
blot haemorrhages
superficial/ flame shaped haemorrhages</td></tr>
<tr><td>R2</td><td>‘Background DR – observable’
Four or more blot haemorrhages in one hemi-field only (Inferior and superior hemi-fields delineated by a line passing through the centre of the fovea and optic disc)</td></tr>
<tr><td>R3</td><td>‘Background DR – referable’
Any of the following features:
Four or more blot haemorrhages in both inferior and superior hemi-fields
Venous beading
IRMA</td></tr>
<tr><td>R4</td><td>‘Proliferative DR’
Any of the following features:
Active new vessels
Vitreous haemorrhage</td></tr>
<tr><td>R6</td><td>‘Not adequately visualised’
Retina not sufficiently visible for assessment</td></tr>
<tr><td colspan="2">MACULOPATHY</td></tr>
<tr><td>M0</td><td>‘No maculopathy’
No features ≤2 disc diameters from the centre of the fovea sufficient to qualify for M1 or M2</td></tr>
<tr><td>M1</td><td>‘Observable maculopathy’
Lesions as specified below within a radius of >1 but ≤2 disc diameters the centre of the fovea:
Any hard exudates</td></tr>
<tr><td>M2</td><td>‘Referable maculopathy’
Lesions as specified below within a radius of ≤1 disc diameter of the centre of the fovea:
Any blot haemorrhages
Any hard exudates</td></tr>
</table>

Trial treatment mailing error

Each LENS trial treatment pack, and the bottles of trial treatment it contained, included a unique pack identifier number (pack ID). Wherever possible, these pack IDs were checked with participants at randomization (in person) and follow up (telephone) assessments to confirm that the correct packs of trial treatment had been mailed to them (with a new pack of randomized trial treatment being mailed to a participant once every 180 days). During a routine follow up telephone assessment in late 2022, it was ascertained that a participant had received a pack of trial treatment with an incorrect pack ID (i.e. a pack ID for trial treatment not assigned to them, but assigned to another participant). Urgent investigation confirmed that there had been an inappropriate reordering of data in 3 mailing order spreadsheets by the drug distribution depot on 2 consecutive days. This error temporarily affected 28 participants in total, all of whom were immediately contacted. At the time of contact, 18 of the 28 participants had started taking trial treatment from the incorrect packs and 10 had not. The error was explained to the affected participants. No SAEs occurred during temporary exposure to trial treatment from the erroneous packs. No participants requested to be unmasked and all members of the research team also remained masked to the assigned treatment arm for these participants. All 28 participants were resupplied with randomized trial treatment.

This error was reported to the Research Ethics Committee and to the Medicines and Healthcare products Regulatory Agency, and it was categorized as a Serious Breach.

A detailed review of the handling of all 808 previous trial treatment mailing orders (including both active run-in and randomized trial treatment) was conducted by the drug distribution depot to confirm that no other errors in handling mailing order spreadsheets had occurred, and all 75 subsequent orders were reviewed until the end of the trial.

It was subsequently confirmed that, of the 28 participants temporarily affected by this error, 14 were mailed trial treatment packs from the appropriate treatment arm and 14 were mailed trial treatment packs from the incorrect treatment arm.

Clarification regarding primary outcome terminology

In Versions 1.0 to 6.3 of the protocol (noting that the trial commenced recruitment with Version 6.0 in place) and in earlier entries of the LENS trial in clinical trial registries (NCT03439345, ISRCTN15073006), the terms referable' and 'clinically significant' terms were used interchangeably to describe the development of a grading of diabetic retinopathy or maculopathy that would be counted as a primary outcome. During the trial, the Steering Committee recognized that this could prove confusing given that 'clinically significant' is a specific description used to categorize advanced macular edema. Therefore, the wording in both the protocol (Version 7.0) and in the clinical trial registries was updated to use consistent terminology by only referring to progression of diabetic retinopathy or maculopathy to a grade consistent with a primary outcome as 'referable' disease (i.e. to avoid the term 'clinically significant').

There was no change to the components of the primary outcome before or during the conduct of the trial i.e. the update referred to above was only a clarification regarding the description of the primary outcome. This can be confirmed in version 1.0 of the protocol which clearly states that a primary outcome of progressive retinopathy or maculopathy was defined according to the Scottish grading scheme as R3 (i.e. referable background diabetic retinopathy) or R4 (i.e. proliferative diabetic retinopathy) or M2 (i.e. referable maculopathy) in at least one eye. The primary outcome therefore remained unchanged throughout the conduct of the trial.

Figure S1. CONSORT diagram of the LENS trial

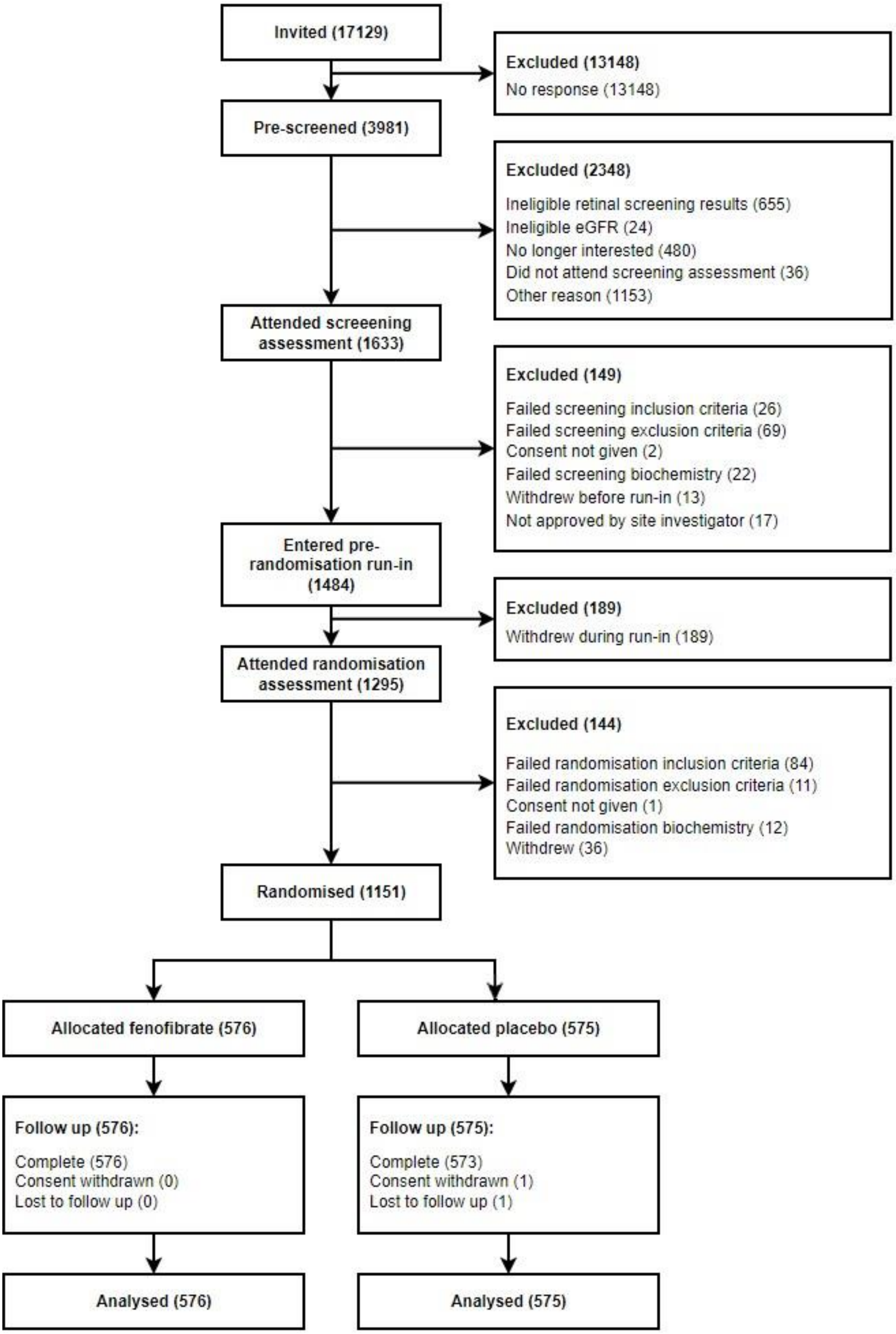

Figure S2. Effect of fenofibrate compared to placebo on eGFR

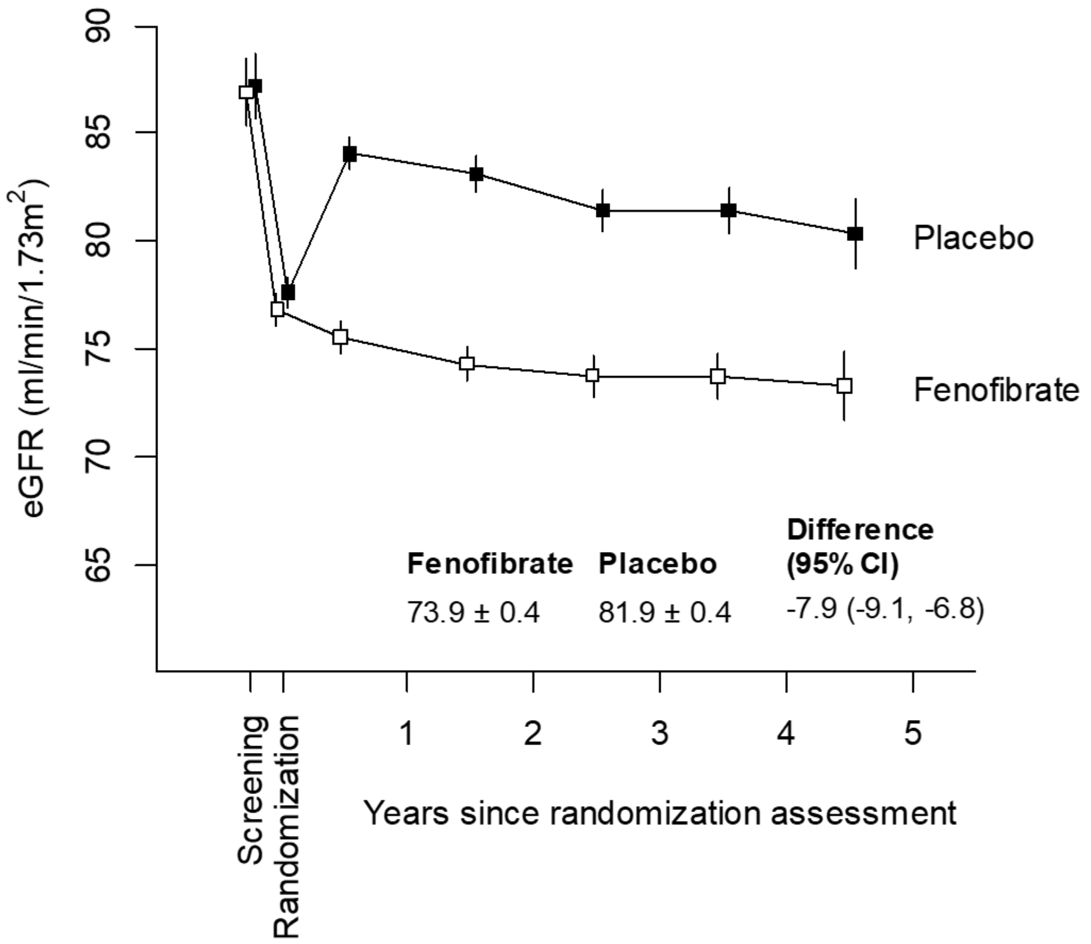

The figure shows mean eGFR at the screening assessment, the randomization assessment and in one−year windows after randomization by treatment allocation.

Linear mixed models for repeated measures (MMRM) analyses were used to estimate the mean eGFR by treatment group at each follow−up time point, as well as a trial−averaged difference in mean follow−up levels, between fenofibrate and placebo. The models adjusted for baseline (screening) eGFR and the randomization minimization criteria (in the same categories used in the minimization process), and assumed an unstructured covariance matrix. Vertical lines indicate the 95% confidence intervals for the estimated means. The coordinates of the boxes are shifted slightly on the x axis to avoid overlap.

Table S1. NHS Scotland's Diabetic Eye Screening Programme grading scheme for retinopathy and maculopathy

Grading	Description	Findings	Outcome
**RETINOPATHY (excluding the macula)**			
R0	No DR anywhere	-	Rescreen in 12-24 months
R1	Mild background diabetic retinopathy	The presence of at least one of any of the following features anywhere:<br>• dot hemorrhages / microaneurysms<br>• hard exudates<br>• cotton wool spots<br>• blot hemorrhages*<br>• superficial/ flame shaped hemorrhages	Rescreen in 12 months
R2	Observable background diabetic retinopathy	Four or more blot hemorrhages* in one hemi-field only (Inferior and superior hemi-fields delineated by a line passing through the centre of the fovea and optic disc)	Rescreen in 6 months
R3	Referable background diabetic retinopathy	Any of the following features:<br>• four or more blot hemorrhages* in both inferior and superior hemi-fields<br>• Venous beading<br>• Intraretinal Microvascular Abnormalities (IRMA)	Specialist referral (routine)
R4	Proliferative diabetic retinopathy	Any of the following features:<br>• Active new vessels<br>• Vitreous hemorrhage	Specialist referral (urgent)
R4i†	Treated proliferative diabetic retinopathy	Any of the following features:<br>• Inactive new vessels with evidence of laser treatment	Rescreen in 12 months
R6	Not adequately visualized	Retina not sufficiently visible for assessment	Technical failure
**MACULOPATHY**			
M0	No maculopathy	No features ≤2 disc diameters from the centre of the fovea sufficient to qualify for M1 or M2	Rescreen in 12-24 months
M1	Observable maculopathy	Lesions as specified below within a radius of >1 but ≤2 disc diameters from the centre of the fovea:<br>• Any hard exudates	Rescreen in 12 months
M2	Referable maculopathy	Lesions as specified below within a radius of ≤1 disc diameter of the centre of the fovea:<br>• Any blot hemorrhages*<br>• Any hard exudates	OCT surveillance scan or Rescreen in 12 months‡

* Blot hemorrhage has the same or greater diameter as a retinal vein crossing the optic disc

† R4i is not counted towards the primary outcome as it represents inactive disease

‡ At the start of LENS in 2018 and until end-2021, all patients in Scotland with M2 retinal screening results required referral to a specialist in ophthalmology. During 2022, the Diabetic Eye Screening programme started to introduce a phased change to the management pathway of patients with M2 disease. In the new pathway, M2 in the context of poor visual acuity (e.g. 6/9.5 or worse) leads to optical coherence tomography (OCT) imaging followed by referral to an ophthalmologist if there is evidence of macular edema.

Table S2. Serious Adverse Events in run-in, categorized by MedDRA system organ class

	Fenofibrate (N=1484)	
Cardiac disorders	4	(0.3%)
Gastrointestinal disorders	4	(0.3%)
General disorders and administration site conditions	2	(0.1%)
Hepatobiliary disorders	1	(0.1%)
Infections and infestations	9	(0.6%)
Injury, poisoning and procedural complications	4	(0.3%)
Metabolism and nutrition disorders	3	(0.2%)
Musculoskeletal and connective tissue disorders	1	(0.1%)
Neoplasms benign, malignant and unspecified (incl cysts and polyps)	1	(0.1%)
Nervous system disorders	1	(0.1%)
Skin and subcutaneous tissue disorders	1	(0.1%)
Surgical and medical procedures	4	(0.3%)
Vascular disorders	1	(0.1%)
**Subtotal: Any Serious Adverse Event**	35	(2.4%)

Figures are number of participants (%).
Only categories with events are listed.

Table S3. Representativeness of the trial population

Category	Comment
Disease, problem, or condition under investigation	Diabetic retinopathy and maculopathy
Special considerations related to:	
Sex	At a given HbA1c level and with similar diabetes duration, sex does not appear to be a key risk factor for the presence or progression of diabetic retinopathy; the worldwide prevalence of visual loss and moderate to severe vision impairment due to diabetic retinopathy is similar in men and women by age.
Age	Given the importance of diabetes duration as a risk factor for diabetic retinopathy, increasing age is associated with higher risks of retinopathy and vision-threatening disease; however people with type 1 diabetes develop diabetes earlier and are at higher risk of progressive retinopathy than people with type 2 diabetes, so they develop significant disease at a younger age than people with type 2 diabetes.
Race or ethnic group	Some studies in developed countries have suggested that people of South Asian or Afro-Caribbean background may be at higher risk of any diabetic retinopathy or vison-threatening diabetic retinopathy than Caucasian people, but it is unclear whether this difference is fully accounted for by differences in key risk factors such as glycemic control.
Geography	National and regional retinal screening programmes remain limited in many parts of the world; the prevalence of vision-threatening diabetic retinopathy is reported to be highest in North Africa and the Middle East, followed by Latin America and the Caribbean, but all regions carry a heavy burden of disease.
Other considerations	Key risk factors for the presence and progression of diabetic retinopathy are longer duration of diabetes and exposure to higher HbA1c levels.
Overall representativeness of this trial	Information regarding participants' sex and date of birth was obtained from NHS Scotland, and ethnicity was self-reported. We have previously demonstrated that LENS trial participants are highly representative of all potentially eligible people in Scotland with regard to age, type of diabetes, grading of retinopathy, HbA1c and kidney function, but that the trial included fewer women and people of non-Caucasian ethnicity than expected based on estimates of national Scottish data (even though potentially eligible people were invited without consideration of sex or ethnicity); in the context of worldwide data for retinopathy and vision-threatening disease, an important limitation of the LENS trial is the small number of participants of non-Caucasian ethnicity.

Table S4. Completeness of Follow up

	Fenofibrate (N=576)		Placebo (N=575)		Total (N=1151)	
**Complete follow up information**	576	(100.0%)	573	(99.7%)	1149	(99.8%)
Final follow up assessment with participant	504	(87.5%)	502	(87.3%)	1006	(87.4%)
Final follow up assessment by medical records/GP	37	(6.4%)	33	(5.7%)	70	(6.1%)
Death before end of final follow up period	35	(6.1%)	38	(6.6%)	73	(6.3%)
Registry data confirming vital status during final follow up period	0	(0.0%)	0	(0.0%)	0	(0.0%)
**Incomplete follow up information**	0	(0.0%)	2	(0.3%)	2	(0.2%)
Full withdrawal of consent	0	(0.0%)	1	(0.2%)	1	(0.1%)
No final follow up assessment (loss to follow up)	0	(0.0%)	1	(0.2%)	1	(0.1%)

Figures are number of participants (%).

Table S5. Frequency of retinal screening after randomization

	Fenofibrate (N=576)	Placebo (N=575)
**Retinal screening episodes**		
Count	1485	1469
Average per participant (SE)	2.58 (0.04)	2.55 (0.04)

Figures are total counts and mean ± standard error

Table S6. Modalities of retinal screening and information on retinal image quality and the use of mydriasis

Modality	Retinal screening episodes	Additional information	Right eye	Left eye
	N		N (%)	N (%)
Routine digital screening	2815	**Image quality***		
		Nerve fibre layer visible	2668 (94.8%)	2654 (94.3%)
		Nerve fibre layer not visible	47 (1.7%)	46 (1.6%)
		Small vessels blurred	25 (0.9%)	26 (0.9%)
		Major arcade vessels just blurred	12 (0.4%)	23 (0.8%)
		Image not gradable/technical failure or no information available about image quality	63 (2.2%)	66 (2.3%)
		**Mydriasis**		
		Mydriasis used	765 (27.2%)	765 (27.2%)
		Mydriasis not used	2050 (72.8%)	2050 (72.8%)
Slit lamp biomicroscopy	143	Mydriasis used	143 (100.0%)	143 (100.0%)
OCT Surveillance†	119	-	-	-

* Image quality in the NHS Scotland Diabetic Eye Screening (DES) programme is rated (from best to worst) as nerve fibre layer visible, nerve fibre layer not visible, small vessels blurred, major arcade vessels just blurred, image not gradable/technical failure; in the case of images being not gradable/technical failure, patients typically then have slit lamp biomicroscopy arranged (and may continue with slit lamp biomicroscopy in the longer term for retinal screening).

† During 2022, DES started to introduce a phased change to the management pathway of patients with referable maculopathy. In the new pathway, referable maculopathy in the context of poor visual acuity (Snellen 6/9.5 or worse) leads to optical coherence tomography (OCT) imaging in the first instance.

Table S7. Adherence to randomized trial treatment

	Fenofibrate (N=576)		Placebo (N=575)		Total (N=1151)	
**Full trial**						
**All regimens**						
Randomization	576 / 576	(100%)	575 / 575	(100%)	1151 / 1151	(100%)
Trial mid-point	495 / 560	(88%)	500 / 561	(89%)	995 / 1121	(89%)
End of trial	421 / 541	(78%)	427 / 535	(80%)	848 / 1076	(79%)
Trial average	88%		89%		89%	
**Daily**						
Randomization	447 / 447	(100%)	448 / 448	(100%)	895 / 895	(100%)
Trial mid-point	344 / 379	(91%)	374 / 421	(89%)	718 / 800	(90%)
End of trial	268 / 329	(81%)	322 / 392	(82%)	590 / 721	(82%)
Trial average	90%		89%		90%	
**Alternate days**						
Randomization	129 / 129	(100%)	127 / 127	(100%)	256 / 256	(100%)
Trial mid-point	151 / 181	(83%)	126 / 140	(90%)	277 / 321	(86%)
End of trial	153 / 212	(72%)	105 / 143	(73%)	258 / 355	(73%)
Trial average	83%		88%		85%	
**Censored at primary outcome**						
**All regimens**						
Randomization	576 / 576	(100%)	575 / 575	(100%)	1151 / 1151	(100%)
Trial mid-point	443 / 498	(89%)	428 / 475	(90%)	871 / 973	(90%)
End of trial	325 / 412	(79%)	303 / 372	(81%)	628 / 784	(80%)
Trial-average	89%		90%		90%	
**Daily**						
Randomization	447 / 447	(100%)	448 / 448	(100%)	895 / 895	(100%)
Trial mid-point	302 / 331	(91%)	316 / 350	(90%)	618 / 681	(91%)
End of trial	198 / 237	(84%)	227 / 270	(84%)	425 / 507	(84%)
Trial-average	91%		91%		91%	
**Alternate days**						
Randomization	129 / 129	(100%)	127 / 127	(100%)	256 / 256	(100%)
Trial mid-point	141 / 167	(84%)	112 / 125	(90%)	253 / 292	(87%)
End of trial	127 / 175	(73%)	76 / 102	(75%)	203 / 277	(73%)
Trial-average	84%		89%		86%	

Adherence at specific time points was calculated as the number of participants taking trial treatment divided by the number of participants who were alive and not fully withdrawn from the trial at that point. Average adherence within each treatment arm and time period of interest was calculated as the sum over all participants of the adherent days divided by the sum over all participants of the days at risk. Data are provided for both regimens (i.e. daily treatment and alternate day treatment) combined and separately.

Table S8. Reasons for stopping randomized trial treatment

	Fenofibrate (N=576)		Placebo (N=575)		P
Serious adverse event (SAE)					
Cardiac disorders	2	(0.3%)	1	(0.2%)	
Gastrointestinal disorders	2	(0.3%)	3	(0.5%)	
Hepatobiliary disorders	3	(0.5%)	4	(0.7%)	
Infections and infestations	1	(0.2%)	1	(0.2%)	
Injury, poisoning and procedural complications	0	(0.0%)	1	(0.2%)	
Investigations	0	(0.0%)	1	(0.2%)	
Neoplasms benign, malignant and unspecified (incl cysts and polyps)	5	(0.9%)	5	(0.9%)	
Nervous system disorders	4	(0.7%)	4	(0.7%)	
Renal and urinary disorders	3	(0.5%)	1	(0.2%)	
Surgical and medical procedures	1	(0.2%)	3	(0.5%)	
**Subtotal: Any SAE**	21	(3.6%)	24	(4.2%)	0.64
Non-serious adverse event (NSAE)					
Eye disorders	2	(0.3%)	2	(0.3%)	
Gastrointestinal disorders	6	(1.0%)	5	(0.9%)	
Hepatobiliary disorders	0	(0.0%)	1	(0.2%)	
Investigations	1	(0.2%)	3	(0.5%)	
Musculoskeletal and connective tissue disorders	3	(0.5%)	2	(0.3%)	
Nervous system disorders	4	(0.7%)	3	(0.5%)	
Pregnancy, puerperium and perinatal conditions	0	(0.0%)	1	(0.2%)	
Respiratory, thoracic and mediastinal disorders	1	(0.2%)	1	(0.2%)	
Skin and subcutaneous tissue disorders	0	(0.0%)	3	(0.5%)	
**Subtotal: Any NSAE**	17	(3.0%)	21	(3.7%)	0.51
Other medical reasons					
Contraindicated medication	0	(0.0%)	2	(0.3%)	
Fall in eGFR	9	(1.6%)	5	(0.9%)	
Unwilling to use contraception	2	(0.3%)	0	(0.0%)	
**Subtotal: Other medical reasons**	11	(1.9%)	7	(1.2%)	0.34

	Fenofibrate (N=576)		Placebo (N=575)		P
Other non-medical reasons					
Patient wishes	29	(5.0%)	30	(5.2%)	
Other reason	54	(9.4%)	42	(7.3%)	
Withdrew consent for further contact	3	(0.5%)	2	(0.3%)	
**Subtotal: Other non-medical reasons**	86	(14.9%)	74	(12.9%)	0.31
**Any reason**	135	(23.4%)	126	(21.9%)	0.54

Figures are number of participants (%).

Only categories with events are listed.

Table S9. Effect of fenofibrate compared to placebo on treatment for diabetic retinopathy or maculopathy

	Fenofibrate (N=576)		Placebo (N=575)		
	N (%)	Events per 100 person-years	N (%)	Events per 100 person-years	HR (95% CI)
Intravitreal injection	9 (1.6%)	0.4	12 (2.1%)	0.5	
Laser photocoagulation	10 (1.7%)	0.5	17 (3.0%)	0.8	
Vitrectomy	0 (0.0%)	0.0	4 (0.7%)	0.2	
**Treatment for diabetic retinopathy or maculopathy**	17 (3.0%)	0.8	28 (4.9%)	1.3	0.58 (0.31-1.06)

Figures are number of participants (%).Confidence intervals have not been adjusted for multiplicity, and should not be used to infer clinical utility.

Table S10. Effect of fenofibrate compared to placebo on visual acuity, quality of life and visual function*

	Fenofibrate			Placebo			
	N	Mean (95% CI)		N	Mean (95% CI)		Difference between groups (95% CI)	
**Visual acuity†‡**								
Baseline	548	0.04	(0.03-0.05)	552	0.03	(0.02-0.04)		
Year 1		0.04	(0.03-0.05)		0.04	(0.03-0.05)	0.00	(-0.01 - 0.02)
Year 2		0.06	(0.05-0.07)		0.05	(0.03-0.06)	0.02	(0.00 - 0.03)
Year 3		0.05	(0.04-0.06)		0.05	(0.04-0.06)	0.00	(-0.01 - 0.02)
Year 4		0.07	(0.06-0.08)		0.06	(0.05-0.07)	0.01	(-0.01 - 0.02)
Year 5		0.07	(0.06-0.09)		0.08	(0.07-0.10)	-0.01	(-0.03 - 0.01)
Trial average follow-up		0.06	(0.05-0.07)		0.05	(0.05-0.06)	0.00	(-0.01 - 0.01)
**EQ-5D-5L index score†§**								
Baseline	573	0.81	(0.80-0.83)	569	0.81	(0.79-0.83)		
Year 2		0.76	(0.75-0.78)		0.76	(0.74-0.77)	0.00	(-0.02 - 0.02)
End of trial		0.74	(0.72-0.75)		0.74	(0.72-0.76)	-0.01	(-0.03 - 0.02)
Trial average follow-up		0.75	(0.73-0.76)		0.75	(0.74-0.76)	0.00	(-0.02 - 0.02)
**EQ-5D-5L visual analogue scale†§**								
Baseline	576	81	(79-82)	574	81	(79-82)		
Year 2		76	(75-78)		76	(75-78)	0	(-2 - 2)
End of trial		74	(73-76)		76	(74-77)	-1	(-3 - 1)

	Fenofibrate		Placebo		
	N	Mean (95% CI)	N	Mean (95% CI)	Difference between groups (95% CI)
Trial average follow-up		75 (74-76)		76 (75-77)	-1 (-2 - 1)
**VFQ-25 composite score†‖**					
Baseline	576	91 (90-92)	575	92 (91-93)	
Year 2		90 (89-91)		90 (90-91)	0 (-1 - 1)
End of trial		89 (89-90)		89 (88-89)	1 (0 - 2)
Trial average follow-up		90 (89-91)		89 (89-90)	0 (-1 - 1)

[*] Shown are arithmetic means and 95% confidence intervals. Confidence intervals have not been adjusted for multiplicity, and should not be used to infer clinical utility.

[†] The estimates were derived from a linear mixed model repeated measures adjusted for the baseline value and the minimization criteria.

[‡] Baseline visual acuity is taken from routine measurement within NHS Scotland’s Diabetic Eye Screening programme (within 18 months of randomization) and with conversion from Snellen to LogMAR where necessary; analysis of the better eye: if only 1 eye, analyse that eye; if 2 eyes, use eye with better acuity; if identical acuity in both eyes, use the right eye.

[§] Quality of life was measured using the EQ-5D-5L instrument, and valued by mapping to the EQ-5D-3L UK value set using the mapping function developed by Hernández Alava et al. PharmacoEconomics (2022). The visual analogue scale is a score out of 100.

[‖] Visual function is based on the Visual Function Questionnaire-25.

Table S11. Effect of fenofibrate compared to placebo on major cardiovascular events and non-traumatic lower limb amputations

	Fenofibrate (N=576)		Placebo (N=575)		
	N (%)	Events per 100 person-years	N (%)	Events per 100 person-years	HR (95% CI)
Major cardiovascular events[*]	45 (7.8%)	2.1	43 (7.5%)	2.0	1.05 (0.69-1.60)
Non-traumatic lower limb amputation[†]	4 (0.7%)	0.2	11 (1.9%)	0.5	0.35 (0.11-1.12)

[*] Composite of any major cardiovascular event (defined as myocardial infarction, stroke, coronary or peripheral revascularization).

[†] Composite of any non-traumatic lower limb amputation (defined as minor amputation [distal to the ankle] or major amputation [through or proximal to the ankle]).

Confidence intervals have not been adjusted for multiplicity, and should not be used to infer clinical utility.

Table S12. Effect of fenofibrate compared to placebo on renal function, lipids, HbA1c and urine albumin creatinine ratio by year and as trial-average*

	Fenofibrate		Placebo		
	N	Mean (95% CI)	N	Mean (95% CI)	Difference between groups (95% CI)
**eGFR – mL/min/1.73m²†**					
Baseline	576	86.9 (85.4-88.4)	575	87.2 (85.7-88.7)	
Year 1		75.3 (74.6-76.1)		83.8 (83.1-84.6)	-8.5 (-9.6 - -7.4)
Year 2		74.1 (73.3-74.9)		82.9 (82.1-83.7)	-8.8 (-10.0 - -7.6)
Year 3		73.6 (72.6-74.5)		81.2 (80.2-82.2)	-7.6 (-9.0 - -6.3)
Year 4		73.5 (72.4-74.6)		81.2 (80.2-82.3)	-7.7 (-9.2 - -6.2)
Year 5		73.1 (71.5-74.7)		80.1 (78.6-81.7)	-7.0 (-9.3 - -4.8)
Trial average follow-up		73.9 (73.1-74.7)		81.9 (81.1-82.7)	-7.9 (-9.1 - -6.8)
**Total cholesterol – mg/dL†**					
Baseline	572	156.4 (153.4-159.4)	567	156.8 (153.6-159.9)	
Year 1		150.8 (148.8-152.8)		157.1 (155.1-159.1)	-6.3 (-9.1 - -3.4)
Year 2		151.4 (148.8-153.9)		158.1 (155.5-160.7)	-6.7 (-10.4 - -3.1)
Year 3		152.2 (149.7-154.6)		157.9 (155.5-160.4)	-5.8 (-9.2 - -2.3)
Year 4		154.6 (151.9-157.3)		155.4 (152.6-158.1)	-0.8 (-4.7 - 3.1)
Year 5		152.8 (147.9-157.6)		154.2 (149.3-159.0)	-1.4 (-8.3 - 5.5)
Trial average follow-up		152.3 (150.4-154.3)		156.5 (154.6-158.5)	-4.2 (-7.0 - -1.4)
**Non-HDL cholesterol – mg/dL†**					

	Fenofibrate			Placebo			
	N	Mean (95% CI)		N	Mean (95% CI)		Difference between groups (95% CI)
Baseline	568	105.8	(103.0-108.7)	560	106.2	(103.1-109.3)	
Year 1		101.3	(98.8-103.8)		107.3	(104.8-109.9)	-6.0 (-9.6 - -2.5)
Year 2		102.4	(100.0-104.8)		108.2	(105.8-110.6)	-5.8 (-9.2 - -2.4)
Year 3		103.4	(101.0-105.9)		108.1	(105.7-110.6)	-4.7 (-8.2 - -1.2)
Year 4		105.7	(103.0-108.4)		104.8	(102.1-107.6)	0.9 (-3.0 - 4.7)
Year 5		103.6	(98.8-108.4)		103.0	(98.2-107.8)	0.6 (-6.2 - 7.4)
Trial average follow-up		103.3	(101.3-105.3)		106.3	(104.3-108.3)	-3.0 (-5.8 - -0.2)
**HDL cholesterol – mg/dL†**							
Baseline	568	50.6	(49.2-51.9)	560	50.4	(49.2-51.6)	
Year 1		49.0	(48.3-49.6)		49.6	(49.0-50.3)	-0.7 (-1.6 - 0.3)
Year 2		48.8	(48.0-49.5)		49.4	(48.7-50.2)	-0.6 (-1.7 - 0.4)
Year 3		48.3	(47.6-49.0)		49.3	(48.6-50.0)	-1.0 (-2.0 - 0.1)
Year 4		48.9	(48.0-49.7)		49.9	(49.1-50.8)	-1.1 (-2.3 - 0.2)
Year 5		48.3	(47.0-49.7)		51.2	(49.8-52.6)	-2.9 (-4.8 - -0.9)
Trial average follow-up		48.6	(48.0-49.2)		49.9	(49.3-50.5)	-1.2 (-2.1 - -0.4)
**Triglycerides – mg/dL†**							
Baseline	571	140.2	(133.5-147.2)	563	136.7	(130.7-143.0)	
Year 1		113.3	(108.9-117.8)		135.8	(130.5-141.4)	-16.6% (-21.2% - -11.8%)
Year 2		115.4	(110.7-120.4)		132.7	(127.0-138.6)	-13.0% (-18.1% - -7.6%)
Year 3		117.6	(113.0-122.4)		137.9	(132.5-143.6)	-14.7% (-19.4% - -9.7%)

	Fenofibrate			Placebo			
	N	Mean (95% CI)		N	Mean (95% CI)		Difference between groups (95% CI)
Year 4		117.3	(111.7-123.2)		135.1	(128.4-142.1)	-13.2% (-19.0% - -6.8%)
Year 5		126.8	(113.3-141.8)		142.4	(127.2-159.4)	-11.0% (-23.9% - 4.2%)
Trial average follow-up		118.0	(113.7-122.4)		136.7	(131.7-142.0)	-13.7% (-18.1% - -9.1%)
**HbA1c – mmol/mol†**							
Baseline	538	66.4	(65.0-67.7)	539	66.3	(65.0-67.7)	
Year 1		66.0	(65.1-66.9)		66.9	(66.0-67.8)	-0.8 (-2.1 - 0.4)
Year 2		66.9	(65.8-68.1)		67.6	(66.4-68.8)	-0.7 (-2.3 - 1.0)
Year 3		66.3	(65.2-67.5)		66.7	(65.6-67.9)	-0.4 (-2.1 - 1.3)
Year 4		67.0	(65.8-68.3)		66.9	(65.6-68.1)	0.2 (-1.6 - 2.0)
Year 5		67.1	(64.8-69.3)		66.1	(63.9-68.4)	0.9 (-2.2 - 4.1)
Trial average follow-up		66.7	(65.8-67.6)		66.8	(65.9-67.8)	-0.2 (-1.5 - 1.2)
**Urine albumin creatinine ratio – mg/g†**							
Baseline	312	14.4	(12.3-16.9)	310	16.6	(14.0-19.6)	
Year 1		11.7	(10.3-13.2)		12.8	(11.2-14.5)	-8.5% (-23.3% - 9.1%)
Year 2		12.0	(10.3-13.9)		15.2	(13.0-17.7)	-21.1% (-36.2% - -2.5%)
Year 3		13.7	(11.9-15.8)		16.9	(14.7-19.6)	-19.1% (-34.1% - -0.8%)
Year 4		14.4	(12.2-16.9)		17.7	(15.0-20.8)	-18.7% (-35.3% - 2.2%)
Year 5		16.9	(13.1-21.9)		15.6	(11.8-20.5)	8.8% (-25.0% - 57.8%)
Trial average follow-up		13.6	(12.1-15.3)		15.5	(13.8-17.5)	-12.4% (-25.8% - 3.5%)

	Fenofibrate		Placebo		
	N	Mean (95% CI)	N	Mean (95% CI)	Difference between groups (95% CI)

[*] Shown are arithmetic means and 95% confidence intervals for eGFR, total cholesterol, non-HDL cholesterol, HDL cholesterol and HbA1c; shown are geometric means and approximate 95% confidence intervals for triglycerides and urine albumin to creatinine ratio.

[†] The estimates were derived from a linear mixed model repeated measures adjusted for the baseline value and the minimization criteria.

Confidence intervals have not been adjusted for multiplicity, and should not be used to infer clinical utility. For triglycerides and the urine albumin to creatinine ratio, the difference reflects a percentage difference. To convert the values for cholesterol to millimoles per liter multiply by 0.02586. To convert the values for triglycerides to millimoles per liter multiply by 0.01129.

Table S13. Serious adverse events after randomization, categorized by MedDRA system organ class

	Fenofibrate (N=576)	Placebo (N=575)
Blood and lymphatic system disorders	5 (0.9%)	2 (0.3%)
Cardiac disorders	37 (6.4%)	36 (6.3%)
Congenital, familial and genetic disorders	0 (0.0%)	0 (0.0%)
Ear and labyrinth disorders	0 (0.0%)	0 (0.0%)
Endocrine disorders	1 (0.2%)	1 (0.2%)
Eye disorders	6* (1.0%)	0 (0.0%)
Gastrointestinal disorders	21 (3.6%)	27 (4.7%)
General disorders and administration site conditions	12 (2.1%)	16 (2.8%)
Hepatobiliary disorders	6 (1.0%)	7 (1.2%)
Immune system disorders	0 (0.0%)	0 (0.0%)
Infections and infestations	49 (8.5%)	50 (8.7%)
Injury, poisoning and procedural complications	23 (4.0%)	28 (4.9%)
Investigations	4 (0.7%)	3 (0.5%)
Metabolism and nutrition disorders	16 (2.8%)	21 (3.7%)
Musculoskeletal and connective tissue disorders	11 (1.9%)	7 (1.2%)
Neoplasms benign, malignant and unspecified (incl cysts and polyps)	29 (5.0%)	23 (4.0%)
Nervous system disorders	33 (5.7%)	27 (4.7%)
Pregnancy, puerperium and perinatal conditions	0 (0.0%)	0 (0.0%)
Psychiatric disorders	6 (1.0%)	5 (0.9%)
Renal and urinary disorders	14 (2.4%)	10 (1.7%)
Reproductive system and breast disorders	2 (0.3%)	1 (0.2%)
Respiratory, thoracic and mediastinal disorders	19 (3.3%)	14 (2.4%)
Skin and subcutaneous tissue disorders	3 (0.5%)	4 (0.7%)
Social circumstances	0 (0.0%)	0 (0.0%)
Surgical and medical procedures	50 (8.7%)	52 (9.0%)
Vascular disorders	3 (0.5%)	4 (0.7%)
Product issues	0 (0.0%)	0 (0.0%)
**Subtotal: Any Serious Adverse Event**	208 (36.1%)	204 (35.5%)

MedDRA = Medical Dictionary for Regulatory Activities.
Figures are number of participants (%).
*Eye disorder SAEs: cataract, vitreous hemorrhage X2, retinal vein occlusion, diabetic glaucoma, retinal hemorrhage
